# Features of the microalga *Raphidocelis subcapitata*: physiology and applications

**DOI:** 10.1007/s00253-024-13038-0

**Published:** 2024-02-19

**Authors:** Manuela D. Machado, Eduardo V. Soares

**Affiliations:** 1 Bioengineering Laboratory - CIETI, ISEP-School of Engineering, Polytechnic Institute of Porto, Rua Dr António Bernardino de Almeida, 431, 4249-015 Porto, Portugal; 2 CEB - Centre of Biological Engineering, University of Minho, Campus de Gualtar, 4710-057 Braga, Portugal; 3 LABBELS – Associate Laboratory, Braga/Guimarães, Portugal

**Keywords:** Biorefinery, Ecotoxicity, Microalga nutrition and cultural conditions, Physiology and phylogeny, Reproduction, *Pseudokirchneriella subcapitata (*= *Selenastrum capricornutum)*

## Abstract

**Abstract:**

The microalga *Raphidocelis subcapitata* was isolated from the Nitelva River (Norway) and subsequently deposited in the collection of the Norwegian Institute of Water Research as “*Selenastrum capricornutum* Printz”. This freshwater microalga, also known as *Pseudokirchneriella subcapitata*, acquired much of its notoriety due to its high sensitivity to different chemical species, which makes it recommended by different international organizations for the assessment of ecotoxicity. However, outside this scope, *R. subcapitata* continues to be little explored. This review aims to shed light on a microalga that, despite its popularity, continues to be an “illustrious” unknown in many ways. Therefore, *R. subcapitata* taxonomy, phylogeny, shape, size/biovolume, cell ultra-structure, and reproduction are reviewed. The nutritional and cultural conditions, chronological aging, and maintenance and preservation of the alga are summarized and critically discussed. Applications of *R. subcapitata*, such as its use in aquatic toxicology (ecotoxicity assessment and elucidation of adverse toxic outcome pathways) are presented. Furthermore, the latest advances in the use of this alga in biotechnology, namely in the bioremediation of effluents and the production of value-added biomolecules and biofuels, are highlighted. To end, a perspective regarding the future exploitation of *R. subcapitata* potentialities, in a modern concept of biorefinery, is outlined.

**Graphical Abstract:**

**Figure:**
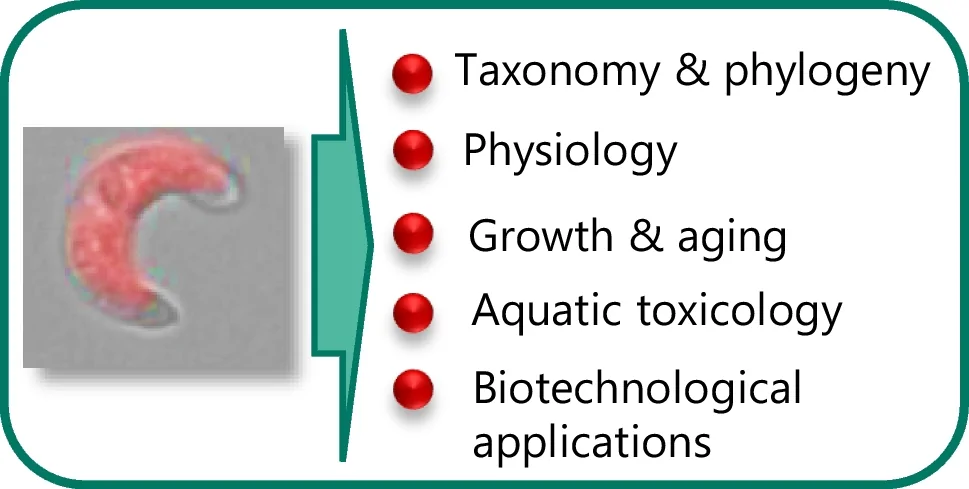

**Key points:**

*• An overview of alga phylogeny and physiology is critically reviewed.*

*• Advances in alga nutrition, cultural conditions, and chronological aging are presented.*

*• Its use in aquatic toxicology and biotechnology is highlighted.*

**Supplementary Information:**

The online version contains supplementary material available at 10.1007/s00253-024-13038-0.

## Introduction

The freshwater unicellular microalga *Raphidocelis subcapitata* (formerly known as *Selenastrum capricornutum* and *Pseudokirchneriella subcapitata*) was originally harvested and isolated from the Nitelva River (Akershus, Norway), in 1959, by the Norwegian researcher Olav Skulberg, and later on deposited in the collection of the Norwegian Institute of Water Research (NIVA) under the code NIVA-CHL 1 (NORCCA 2023a).

*R. subcapitata* is part of the freshwater phytoplankton, being found in ponds, lakes, pools, and rivers. Its presence has been widely reported, all over the world, namely in the USA (Fawley et al. 2006), India (Padmakumar and Tharavathy 2020), Israel, Kazakhstan (Barinova et al. 2009), Czech Republic (Stirk et al. 2013), Spain (Cambra-Sánchez et al. 1998), Romania (Cărăus 2002), between others.

This alga was first employed, in the 60s of the twentieth century, in the evaluation of freshwater eutrophication, in Europe (Skulberg 1964) and the USA (US-EPA 1971; Miller et al. 1978). *R. subcapitata* is considered a representative of both eutrophic and oligotrophic freshwater environments being a very sensitive microorganism to different pollutants. In the 70s–80s, this strain became rapidly popular as a model alga in the evaluation of ecotoxicity, as soon as such assays were developed (Blaise and Vasseur 2005). Currently, *R. subcapitata* is a famous experimental organism, worldwide used, being recommended by several international organizations, such as the American Society for Testing and Materials (ASTM), International Organization for Standardization (ISO), Organization for Economic Co-operation and Development (OECD), and United States Environmental Protection Agency (US-EPA) (OECD 2011; ISO 2012; US-EPA 2012; ASTM 2021) as a bioassay to assess the toxicity of chemicals and evaluation of water quality.

In more recent years, several works have been published reporting the role of *R. subcapitata* in environmental and industrial biotechnology. Examples of this are the bioremediation of industrial effluents (Ribeiro et al. 2022; Fernández-Rodríguez et al. 2022) and pharmaceutical compounds of emerging concern (Hom-Diaz et al. 2022), the production of biofuels (such as third-generation biodiesel) or of compounds that can be used as natural dyes and antioxidants, or to obtain innovative functional food products (Nascimento et al. 2020).

A search in the Web of Science-Clarivate Analytics database (Web of Science Core Collection, All editions), search from 1900 to the present (10 January 2024), using the topics “*Raphidocelis subcapita*ta and review”, “*Pseudokirchneriella subcapitata* and review”, or “*Selenastrum capricornutum* and review”, did not give any review paper on this microalga. Therefore, as far as we know, besides the scientific review published by US-EPA, in the late 70s of the last century (Leischmann et al. 1979), this is the first review paper dedicated to this freshwater microalga.

The present work reviews the taxonomy, phylogeny, ultra-structure, and reproduction of *R. subcapitata*. Alga nutrition, cultural conditions, chronological aging, maintenance, and culture collections where the strain can be found are summarized and critically reviewed. In addition, applied aspects associated with *R. subcapitata* are highlighted, such as those related to aquatic toxicology and environmental and industrial biotechnological applications from the perspective of the modern biorefinery concept. Finally, the future trend of the use of *R. subcapitata* is presented.

## Taxonomy and phylogeny

The phylum *Chlorophyta* (green algae) is ubiquitous in aquatic (primarily in freshwater, 90%) and in some terrestrial habitats, having played a central role in the global ecosystem for hundreds of millions of years (Leliaert et al. 2012). Green algae present a set of distinct features, namely they contain pigments in the chloroplast, similar to those of higher plants (chlorophylls* a* and *b*), accessory pigments (such as carotenes and xanthophylls), and the ability to form starch (as reserve polysaccharide) within the chloroplast, in association with pyrenoids, when present. Plastids descended from a common prokaryotic ancestor (Lee 2018). Five classes were recognized within this phylum: *Chlorophyceae*, *Ulvophyceae*, *Trebouxiophyceae*, *Chlorodendrophyceae*, and *Pedinophyceae* (Marin 2012; Fučíková et al. 2014a; Lemieux et al. 2014). *Chlorophyceae* are a large, ecologically, morphologically, and genetically diverse class. Based on molecular and ultrastructural data, this class comprises five main orders, namely *Sphaeropleales*, *Chlamydomonadales* (*Volvocales*), *Chaetophorales*, *Chaetopeltidales*, and *Oedogoniales* (Leliaert et al. 2012). On the other hand, within *Chlorophyceae*, the large order of *Sphaeropleales* comprises several families, namely *Sphaeropleaceae*, *Selenastraceae*, *Scenedesmaceae*, *Hydrodictyaceae*, *Neochloridaceae*, and *Radiococcaceae* (Tippery et al. 2012), which contain some of the most common planktonic freshwater algae (Leliaert et al. 2012). The *Selenastraceae* family contains among the most promising groups of algae for biotechnological purposes (Yee 2016). This family comprises algae commonly found in freshwaters, generally solitary or colonial, exhibiting a high morphology diversity: fusiform, sickle, croissant, half-moon, or bean shape. Algae belonging to this family reproduce exclusively by autospores and do not have flagellated stages, and chloroplasts can contain starch-covered or naked pyrenoids (Krienitz et al. 2001; Fawley et al. 2006; Fučíková et al. 2014b). Based on molecular studies, different genera were assigned to this family, namely *Ankistrodesmus*, *Kirchneriella*, *Monoraphidium*, *Raphidocelis*, and *Selenastrum* (Krienitz and Bock 2012). Probably one of the most famous members of the *Selenastraceae* family is the alga *S. capricornutum* NIVA-CHL 1.

The strain NIVA-CHL 1 was originally named *S. capricornutum* (Printz) (Blaise and Vasseur 2005). However, over the last few decades, it has undergone several taxonomic changes, having been included in the genera *Raphidocelis* Hindák, *Kirchneria* Hindák, and *Pseudokirchneriella* Hindák (Krienitz et al. 2011). In 1987, Nygaard et al. renamed the strain as *R. subcapitata* and, later on, Hindák, in 1990, designated it *P. subcapitata* (Korshikov) Hindák (Blaise and Vasseur 2005). Subsequently, taking into account phylogenetic studies, the NIVA-CHL 1 strain was placed, again, in the genus *Raphidocelis*, being designated *R. subcapitata* (Korshikov) Nygaard, Komárek, Kristiansen, & Skulberg 1987 (Krienitz et al. 2011). Although the most recent scientific publications refer to this alga as *R. subcapitata*, the strain is usually known, in Europe and North America, as *P. subcapitata* since this is the designation that occurs in standards and guidelines, such as ISO, OECD, and US-EPA (OECD 2011; ISO 2012; US-EPA 2012)*.* In addition, the original name *S. capricornutum* continues to appear in some culture collections such as in the Culture Collection of Algae at the University of Texas at Austin (UTEX), USA (Table 1). To summarize, the current taxonomical position of *R. subcapitata* is as follows (Guiry and Guiry 2015):

- Empire: Eukaryota
- Kingdom: Plantae
- Subkingdom: Viridiplantae
- Phylum: *Chlorophyta*
- Class: *Chlorophyceae*
- Order: *Sphaeropleales*
- Family: *Selenastraceae*
- Genus: *Raphidocelis*

**Table 1:** Examples of culture collections where the standard strain can be found

Culture collection	Strain designation	Code	Country	Reference
American Type Culture Collection (ATCC)	*Pseudokirchneriella subcapitata*	22,662	USA	(ATCC 2023)
Australian National Algae Culture Collection (ANACC)	*Raphidocelis subcapitata*	CS-327	Australia	(ANACC 2023)
Canadian Phycological Culture Centre (CPCC)	*Pseudokirchneriella subcapitata*	37	Canada	(CPCC 2023)
Culture Collection of Algae and Protozoa (CCAP)	*Raphidocelis subcapitata*	278/4	UK	(CCAP 2023b)
Culture Collection of Algae at Gottingen University (Sammlung von Algenkulturen, SAG)	*Raphidocelis subcapitata*	61.81	Germany	(SAG 2023b)
Culture Collection of Algae at the University of Texas at Austin (UTEX)	*Selenastrum capricornutum*	1648	USA	(UTEX 2023a)
Culture Collection of Autotrophic Organisms (CCALA)	*Raphidocelis subcapitata*	433	Czech Republic	(CCALA 2023)
Microbial Collection at the National Institute for Environmental Studies (NIES)	*Raphidocelis subcapitata*	35	Japan	(NIES 2023)
Norwegian Culture Collection of Algae (NORCCA)	*Raphidocelis subcapitata*	NIVA-CHL 1	Norway	(NORCCA 2023a)

The sequencing of nuclear, mitochondrial, and plastid genomes of *R. subcapitata* was carried out. The nuclear genome is composed of 51 Mbp, encoding to 13,383 proteins, being the smallest in the *Sphaeropleales* order. A comparative analysis showed that *R. subcapitata* shares most of its genes with other algae belonging to the *Sphaeropleales* order, which indicates that gene repertoire was conserved in this order. The phylogenetic analysis based on plastid genome sequences indicated that *R. subcapitata* is located in the most basal lineage in the family *Selenastraceae*. The analysis of the mitochondrial genome revealed a dynamic evolutionary history with intron expansion in the *Selenastraceae* family (Suzuki et al. 2018).

## Culture collections

The strain recommended by ASTM, ISO, OECD, and US-EPA for being used in toxicity assays can be obtained in different algal repositories around the world, under the designation of *R. subcapitata*, *P. subcapitata*, or *S. capricornutum* as detailed in Table 1. The strain found in these collections is derived from the original one NIVA-CHL 1. These culture collections provide the alga generally in axenic form (i.e., free of bacteria and other microorganisms), in liquid culture (CCAP 2023a; NIES 2023; NORCCA 2023b; UTEX 2023a), on agar slant (CCAP 2023a; UTEX 2023a), or as dried pellet in an ampoule (freeze-dried) (ATCC 2023).

## Morphology

### Shape

**Fig. 1:**
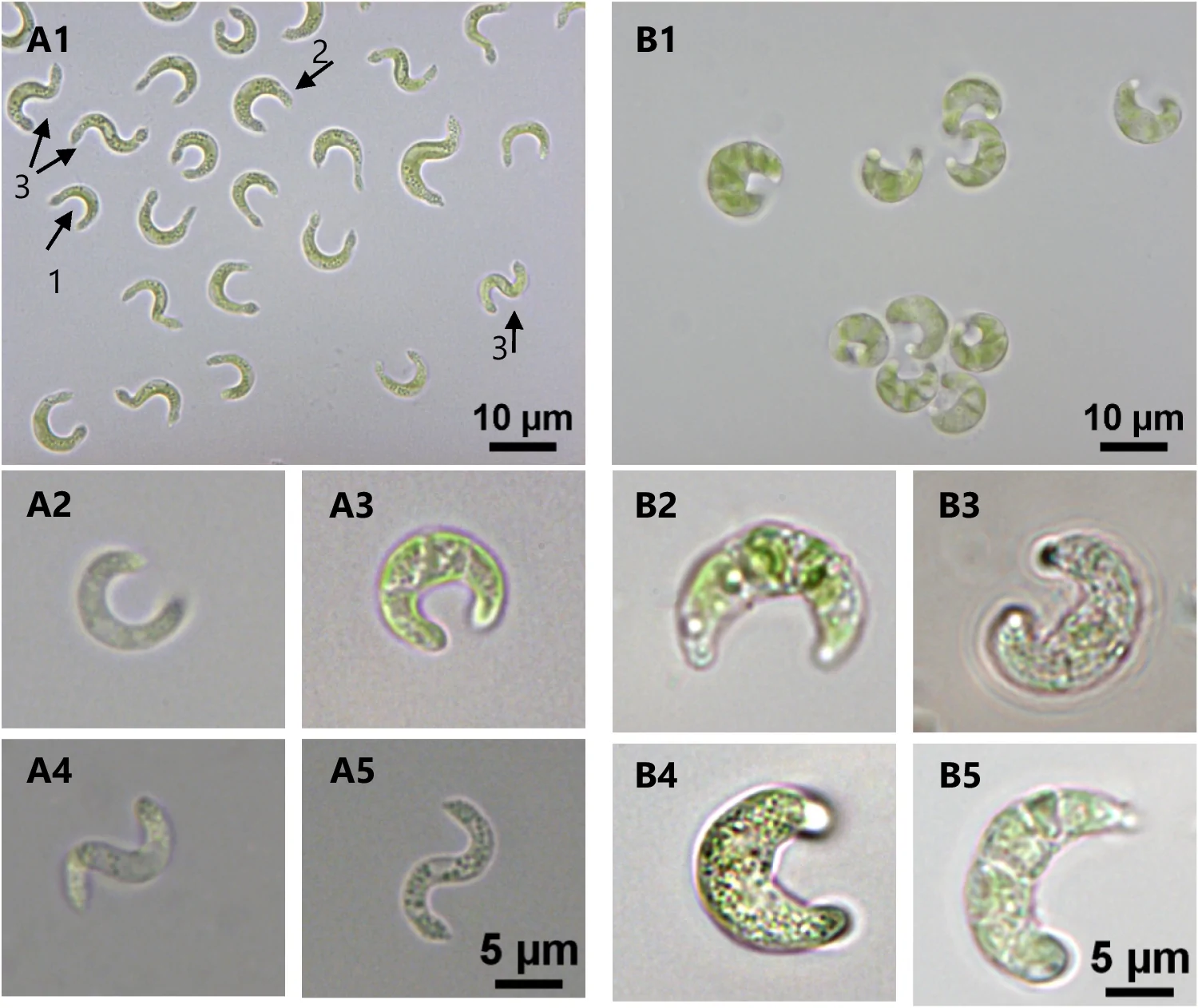
Diversity of the freshwater microalga *R. subcapitata* morphology. A1—different shapes observed during growth; arrows 1 and 2: C-shaped/lunate forms with smaller and larger biovolumes, respectively; arrow 3: helically twisted forms. A2 to A5—higher magnification of algae with C-shaped/lunate form (A2—smaller biovolume; A3—larger biovolume) or with helically twisted form (A4 and A5). B1—algae with an increased biovolume/deformed shape, as a consequence of the exposure for 72 h to 200 µg L^-1^ metolachlor (MET). B2 to B5—higher magnification of algae with an increased biovolume (B2), with a “French croissant”-type shape (B3) or with a deformed morphology and increased mean cell volume (B4 and B5); cells were exposed for 72 h to 200 µg L^-1^ MET (B2, B3, and B5) or 200 µg L^-1^ Cd (B4)

As mentioned above, the unicellular alga *R. subcapitata* was formerly named *Selenastrum* (from the Greek *selene*, “moon” + *astron*, “star”) as a result of its half-moon shape. Different words have been used to describe the morphology of this microalga such as crescent, half-moon, lunate, sickle, bean, C-shaped, and helically twisted (Krienitz et al. 2011; Borges 2016); some shapes of this alga can be seen in Fig. 1; A1 to A5.

This alga is usually described as non-polymorphic; i.e., it retains the same shape during its cell cycle (Blaise and Vasseur 2005; Chèvre and Gregorio 2013). However, a fine analysis of alga morphology, during prolonged culture in OECD medium, revealed that the population in exponential growth (“young” cells) prevalently presented a lunate shape (Fig. 1; A2 and A3), while the cells in the death phase (“aged” cells) predominantly displayed a helically twisted shape (Fig. 1; A4 and A5) (Machado and Soares 2022). Krienitz et al. (2011) also reported that older cells exhibited a helically twisted shape. Furthermore, an alteration from the typical alga shape (lunate) to “French croissant”-type (Fig. 1; B3) or even an aberrant morphology with increased size/biovolume (Fig. 1; B4 and B5), was reported when the alga has been exposed to inorganic or organic pollutants, as detailed in the section “Dry weight and size/biovolume”.

### Arrangement

*R. subcapitata* is mainly present in a solitary form, but can also occur in a colonial form (Krienitz et al. 2011; Borges 2016). A multinucleated form, resembling a palmelloid-like morphology, has been described upon exposure of the alga to toxics; please see section “Formation of a palmelloid-like phenotype”.

The formation of cell aggregates in this unicellular alga is uncommon, which is an advantage since it allows its easy quantification by microscopy or via electronic particle counters (Blaise and Vasseur 2005).

### Dry weight and size/biovolume

*R. subcapitata* presents a cell dry weight of 2–3 × 10^−8^ mg cell^−1^ (OECD 2011).

The individual cells present a length that can vary between 8 to 15 µm and a width between 1.9 and 4 µm. Examples of values reported in the literature are (length × width) 10 × 2 µm (Pollio et al. 1993), 10–15 × 2–4 µm (Blaise and Vasseur 2005), 8–14 × 2–3 µm (OECD 2011), and 8–11 × 3–4 µm (Machado and Soares 2014).

Algal biovolume can be measured with an electronic particle counter (Liu et al. 2023) or using microscopic measurements and appropriate mathematical equations (Machado and Soares 2014). The values described in the literature for *R. subcapitata* biovolume can vary between 22 and 74.5 µm^3^; examples found in the literature are 22 µm^3^ (Machado and Soares 2021a; Ostovich et al. 2023), 40–60 µm^3^ (Blaise and Vasseur 2005), 62 µm^3^ (Patil et al. 2007), and 74.5 µm^3^ (Weiner et al. 2004). Reasons for this wide range of values include different techniques used in biovolume determination and cell volume variation according to alga physiological state. About the last aspect, some authors reported a reduction of the alga size and thinning during the exponential growth phase where the cells display a biovolume of 15–20 µm^3^; in the stationary phase, cells are larger and wider presenting a biovolume from 60–70 µm^3^ (Blaise and Vasseur 2005). However, the opposite was also observed; that is, the alga *R. subcapitata* presents a higher average biovolume during the exponential phase of growth, which decreases in the stationary phase (Machado MD and Soares EV, unpublished results). Yamagishi et al. (2017) also reported a lower average cell diameter in the stationary growth phase (~ 4.4 µm), compared to that measured in the exponential growth phase (4.92–5.00 µm).

#### Cell biovolume as a marker of toxicity

Cell biovolume can be used as a marker of toxicity, as the exposure of *R. subcapitata* to toxics can induce their modification. A cell shrinkage was observed when the alga was exposed to low concentrations of cadmium (Cd), chromium (Cr), copper (Cu), and zinc (Zn) (Machado and Soares 2014). On the contrary, swelling of the cells, as shown by the increase of the mean cell volume, was observed when *R. subcapitata* was exposed to certain chemicals (inorganic or organic) such as Cd (Machado and Soares 2014), Cu (Franklin et al. 2001), nickel oxide (NiO) nanoparticles (Sousa et al. 2018), lithiated cobalt oxide nanosheets (Ostovich et al. 2023), erythromycin (Machado and Soares 2019a), triclosan (TCS) (Machado and Soares 2021a), or metolachlor (MET) (Machado and Soares 2020), particularly at high concentrations (Fig. 1; B1 to B5). For example, *R. subcapitata* exposed to heavy metals, herbicides (MET), or biocides (TCS) can exhibit a biovolume of 200–500 µm^3^; this huge increase in *R. subcapitata* biovolume is particularly evident when alga growth is arrested due to the action of toxics (Machado and Soares 2020, 2021a).

## Cell ultra-structure

### Cell envelope

*R. subcapitata* is a non-motile alga, without known flagellated stages (Miller et al. 1978). The alga presents a rigid cell wall, usually without ornamentation or a mucilaginous cover (Krienitz et al. 2011). However, incrustations in the cell wall can also be present (Fawley et al. 2006). The cover, if present, is thin and smooth containing acidic polysaccharides (Krienitz et al. 2011). When observed by transmission electron microscopy (TEM), *R. subcapitata* cell wall is composed of a single and homogeneous electron-dense layer (Pollio et al. 1993). It is much more difficult to break than the cell wall of *Chlamydomonas reinhardtii* probably due to the cellulose content (Lavoie et al. 2009).

### Chloroplast and pyrenoid

**Fig. 2:**
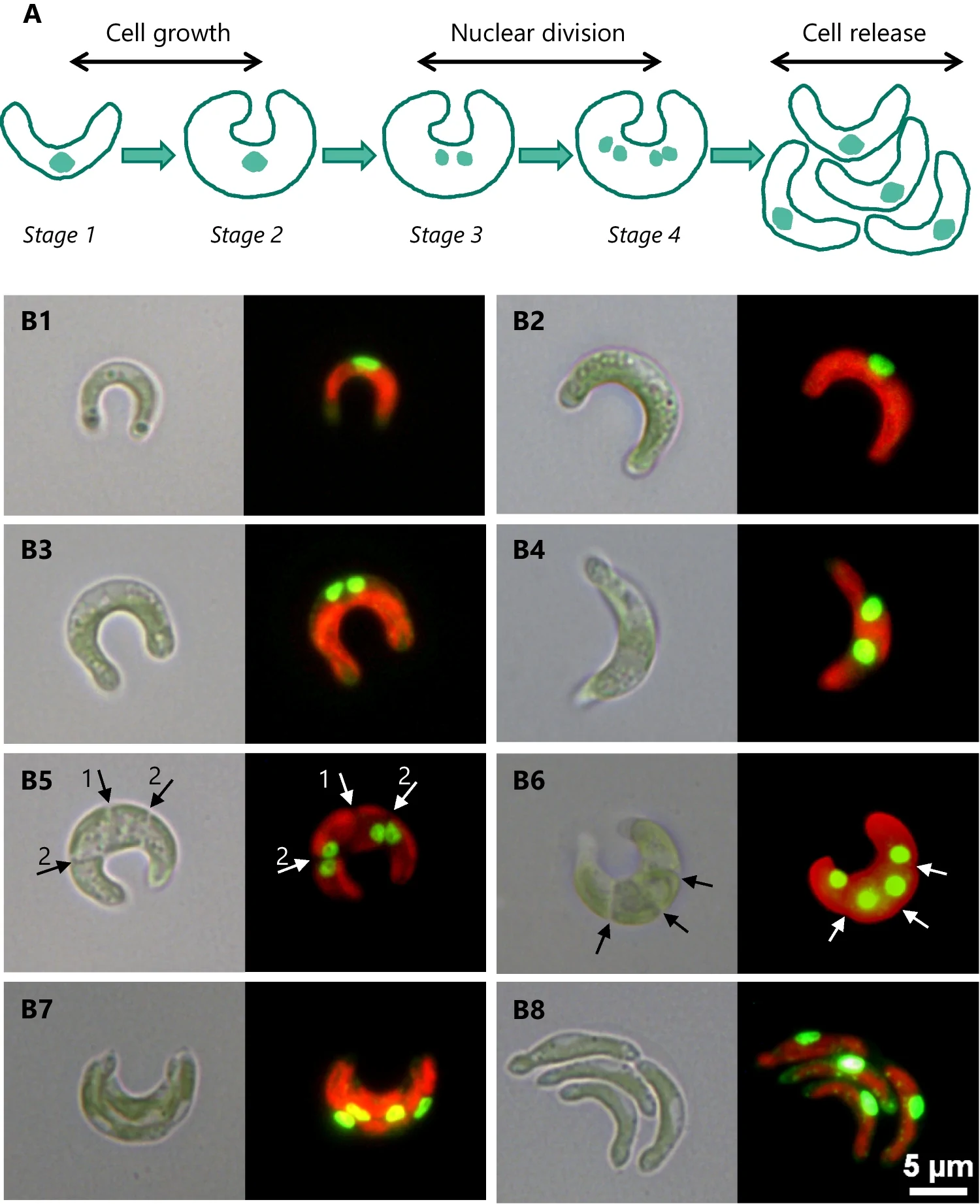
Asexual reproduction of *R. subcapitata* via autospore formation. A—representation of the alga cell cycle; adapted from Machado and Soares (2014). B1—mother cell with a nucleus in a central position. B2—increase in cell size/biovolume. B3—first nuclear division. B4—nuclei separation, after division. B5—second nuclear division and cytokinesis with septum formation and chloroplast constriction; primary cleavage furrow (arrow 1) and secondary cleavages (arrow 2). B6—septa (arrows), perpendicular to the longest axis of the cell, formed between all four nuclei. B7—modification of autospores position in a longitudinal direction (serial arrangement of autospores) inside the parental envelope. B8—release of the autospores. Left and right-side images correspond to the observation of cells by bright field or by fluorescence, respectively. Nuclei (green spots) visualization after cell staining with SYBR Green; the chloroplast can be observed due to its characteristic orange autofluorescence

In *R. subcapitata*, most of the cellular volume is occupied by a -single-large-parietal chloroplast (Fig. 2B1); the remaining cytoplasm occupies the central part of the cell (Pollio et al. 1993).

Pyrenoid can be found in chloroplasts of many algae. This sub-cellular compartment contains the densely packed photosynthetic enzyme ribulose-1,5-bisphosphate carboxylase/oxygenase (RuBisCO) and has a function of increasing the CO_2_ concentration around the RuBisCO (Giordano et al. 2005). The presence or absence of pyrenoid and its structure were used in the past as criteria to distinguish the genera in the *Selenastraceae* family (Krienitz et al. 2001). This structure was found in all selenastracean strains investigated (Krienitz et al. 2011). Usually, naked pyrenoid, i.e., not covered by a regular starch envelope, is not detectable by light microscopy, unless it was used in differential interference contrast optics or specific staining (Krienitz et al. 2001). In *R. subcapitata*, pyrenoid can be seen by TEM, being located in one of the alga apices, with a spherical or irregular shape, and naked sub-cellular structure (Krienitz et al. 2011). The presence of pyrenoid seems to be dependent on cultural conditions and the cell cycle stage of the algae (Meyer et al. 2017).

### Other organelles and cellular inclusions (material reserve compounds)

The cytoplasm of *R. subcapitata* is ribosomes-rich and holds typical eukaryotic organelles, namely, dictyosome, endoplasmic reticulum, a single pleomorphic mitochondrion (often adjacent to the chloroplast), and many small vacuoles (Pollio et al. 1993). The nucleus is located in a central region of the cell (Fig. 2B1).

*R. subcapitata* accumulates reserve material, namely starch and neutral lipids. A recent work showed that young cells (in the exponential phase of growth) produce starch as a reserve material, while aged cells (in the stationary phase) presented lower starch accumulation and a higher level of neutral lipid globules (Machado and Soares 2022). Starch grains are positioned close to the border of the pyrenoid matrix (Krienitz et al. 2011). Stress conditions, namely nitrogen and phosphorous limitations, influence the accumulation of material reserve compounds, such as starch and lipids, as it is detailed in the section “Biotechnological applications”.

## Reproduction

### Sequence of events

*R. subcapitata* reproduces exclusively asexually by autospores (Krienitz et al. 2011; Krienitz and Bock 2012). The cell cycle comprises growth of the parental cell, two nuclear divisions, and release of daughter cells. Four stages can be found in algal population (Fig. 2A); cells in stage 4 showed a mean biovolume ~ 4 times greater than in stage 1 (Machado and Soares 2014). Usually, four daughter cells are formed, with the same shape as the parent cell (autospores), per sporangium.

The mother cell presents the nucleus in the center of the cell (Fig. 2B1) and increases in size (Fig. 2B2) before the first nuclear division (Fig. 2B3), which is followed by septum formation (cleavage furrow) and cytokinesis. After division, the daughter nuclei move away from each other (Fig. 2B4). During the formation of the autospores, a second nuclear division occurs as well as the chloroplast constriction and the division of the protoplast around the four nuclei (Fig. 2B5). It is possible to observe the presence of septa, perpendicular to the longest axis of the cell: the primary septum after the first nuclear division and secondary septa (Fig. 2B5, arrows 1 and 2, respectively) formed between all four nuclei after the second nuclear division (Fig. 2B5 and B6). Subsequently, the new four cells modify their position, in a longitudinal direction—serial arrangement of autospores (Fig. 2B7). The rupture of the parent cell wall (sporangium) allows the release of the four autospores (Fig. 2B8) (Krienitz et al. 2011; Krienitz and Bock 2012; Machado and Soares 2014).

During the exponential growth phase, the *R. subcapitata* population is composed mainly (~ 90%) of cells containing one (1) nucleus. The percentage of this type of cell increases over time; in the stationary phase, the population is almost entirely composed (≥ 99%) of cells containing one (1) nucleus (Machado and Soares 2022).

### Cell cycle checkpoints

The cell cycle of eukaryotic organisms has different control points. One of them, in unicellular algae, is associated with reaching a critical cell volume (Vítová and Zachleder 2005). For instance, in *Chlorella vulgaris* (Rioboo et al. 2009) and *C. reinhardtii* (Matsumura et al. 2003), algae grow during G1 phase until reaching a critical threshold size, after which the algae begin to divide. Similarly, in *R. subcapitata*, an increase in cell size is observed (Fig. 2B2) before the first nuclear division.

Because nuclear and cell divisions are temporally disconnected during the progression of the reproductive cycle, two or more checkpoints can be observed. In fact, the cell cycle of *R. subcapitata* is arrested at stage 2 (before the first nuclear division) when exposed to Cu(II) ions or NiO nanoparticles or stage 4 (before the release of daughter cells) when incubated with Cd(II) (Machado and Soares 2014; Sousa et al. 2018). This means that cell cycle analysis can constitute an alternative endpoint in the assessment of ecotoxicity, as referred to in the section “Elucidation of mode of action of toxics”.

## Formation of a palmelloid-like phenotype

**Fig. 3:**
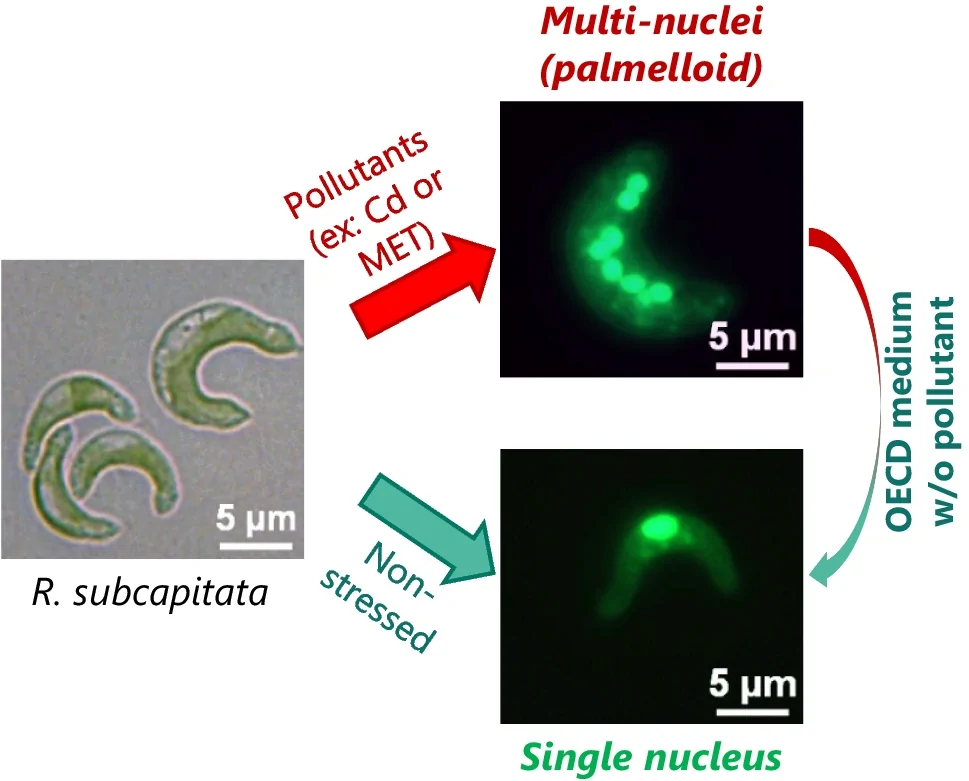
Palmelloid-like morphology in *R. subcapitata*. Certain inorganic and organic pollutants, such as Cd or metolachlor (MET), induce in *R. subcapitata* a palmelloid-like phenotype: forming of clusters of cells (multi-nuclei state) confined by the parental cell wall. This phenotype can be seen as an adaptive strategy of the alga to manage stress. The alga reverts to a unicellular lifestyle (single-nucleus state), with normal cell size/biovolume and shape, upon being transferred to a fresh culture medium without pollutants

When exposed to toxics, such as heavy metals (Cd or Cr) or organic compounds (MET), *R. subcapitata* population contains multiple nuclei (six, eight, or even more) inside the parental envelope (Machado and Soares 2014, 2020; Yamagishi et al. 2017). The exposure to Cd or MET, at environmentally relevant concentrations that arrest growth, originates a population composed mainly of living cells, in a multinucleated state, i.e., containing four (4) or more nuclei, accompanied by the accumulation of neutral lipids (Machado and Soares 2023). This means that under specific stress conditions, *R. subcapitata* undergoes repeated mitosis rounds without the rupture of the wall envelope and the release of the new cells, which provokes the increase of the size of the parental cell and the forming of clusters of cells confined by the parental cell wall (Fig. 3), originating a phenotype that resembles the palmelloid-like morphology (Machado and Soares 2023), observed in algae belonging to the genus *Chlamydomonas* (de Carpentier et al. 2019), *Chlorella* (Fisher et al. 2016), *Dunaliella* (Borowitzka and Siva 2007), and *Scenedesmus* (Lürling and Van Donk 1997), when exposed to abiotic or biotic stress. The switch from a single-nucleus state to a palmelloid-like phenotype can be viewed as an adaptive response to manage/survive stress (Borowitzka 2018; de Carpentier et al. 2019), which appears to be conserved in green algae.

In *R. subcapitata*, the formation of a palmelloid-like phenotype seems to be a specific cellular response to stress, dependent on the mode of action of the pollutant, its concentration, and exposure time. The multinucleated state of the cell population is a transitory and a reversible phenotype, since the *R. subcapitata* population reverts to a unicellular lifestyle, with normal cell biovolume and shape (Fig. 3), upon being transferred to a fresh culture medium without pollutants (Machado and Soares 2023).

## Alga nutrition

### Carbon source

*R. subcapitata*, as an autotrophic organism, carries out photosynthesis, using light as an energy source and CO_2_ as a carbon source. However, it is reported that this alga can also grow under heterotrophic conditions, i.e., without photosynthesis, using glucose as a carbon source (Suzuki et al. 2018; Priyanka et al. 2020). Glucose supplementation (5 or 10 g L^−1^) results in an increase in biomass and lipid production, and depletion in chlorophyll content (Suzuki et al. 2018; Priyanka et al. 2020).

For autotrophic growth, to ensure adequate carbon (C), the culture medium contains sodium hydrogencarbonate (OECD 2011; US-EPA 2012).

### Nitrogen source

*R. subcapitata* is able to assimilate and use ammonium (NH_4_^+^) and nitrate (NO_3_^−^) as a nitrogen (N) source. These two chemical species are present in OECD and US-EPA culture media (OECD 2011; US-EPA 2012). US-EPA limits N concentration to values ≤ 10 mg L^−1^ in culture medium intended for algae growth for toxicity assessment tests (US-EPA 2012).

Nitrogen source can influence algal growth and biomass productivity or even promote physiological alterations in the alga due to different affinities to NH_4_^+^ or NO_3_^−^ (Lachmann et al. 2019). Silva et al. (2015) report that *R. subcapitata* has, as expected, a preference for NH_4_^+^ since, after being assimilated, it is directly used as N source by the alga, while NO_3_^−^ needs to be previously reduced before N can be incorporated into the microalga biomolecules.

### Phosphorous source

In the formulation of the culture media generally used for the growth of *R. subcapitata*, phosphorous (P) is supplied in the form of phosphate (PO_4_^3−^), in a concentration of 0.57 mg L^−1^ and 0.87 mg L^−1^, in OECD and US-EPA media, respectively (OECD 2011; US-EPA 2012).

### Trace elements requirement

In addition to macronutrients, *R. subcapitata* also requires small amounts of micronutrients, such as Cu, iron (Fe), manganese (Mn), molybdenum (Mo), and Zn, for healthy growth.

Cu is involved in algal metabolic processes and is of crucial importance in the photosynthetic electron transport chain and as an enzymatic cofactor (Rocha et al. 2021). It is added to US-EPA and OECD media in concentrations of 0.07 and 0.06 nmol L^−1^, respectively (OECD 2011; US-EPA 2012). Zn makes part of many enzymes and is required for photosynthesis and energy storage (El-Agawany and Kaamoush 2023). This micronutrient is present in US-EPA and OECD media at concentrations of 24 and 22 nmol L^−1^, respectively (OECD 2011; US-EPA 2012). Concentrations of Cu and Zn one order of magnitude higher (1.3 µmol L^−1^ Cu and 2.5 µmol L^−1^ Zn) inhibit 90% of the growth of *R. subcapitata* and adversely alter photosynthetic pigments. The two metals decrease *R. subcapitata* metabolic and photosynthetic activity and affect mitochondrial function (Machado et al. 2015; Filová et al. 2021). The perturbation in photosynthesis contributes to an increased accumulation of reactive oxygen species (ROS) (Machado et al. 2015) and consequent induction of lipid peroxidation and activation of the defense mechanisms with augmented glutathione content (Filová et al. 2021).

Fe is an essential micronutrient in the photosynthetic process, namely in chlorophyll synthesis, and is associated with the activity of electron transport chains (Guerinot and Yi 1994). This trace element is present in the alga growth medium at a concentration of 0.3–0.6 µmol L^−1^ (OECD 2011). An excessive Fe concentration (50-fold higher than normally present in culture medium formulation) promotes a 50% growth inhibition of *R. subcapitata* (Arbildua et al. 2017).

Mn is another important micronutrient in chlorophyll synthesis, which is added to culture media at around 2 µmol L^−1^ Mn (OECD 2011; US-EPA 2012). This element is also part of the oxygen-evolving complex of photosystem II (PSII), acts as an enzymatic cofactor, and intervenes in electron transfer reactions (Quigg 2008).

Nitrogen fixation and NO_3_^−^ reduction are promoted by Mo, which is usually in culture media at ~ 30 nmol L^−1^ (OECD 2011); this element acts as a cofactor of several enzymes present in these processes (Quigg 2008).

## Commonly used culture media

### Chu, Bold’s basal, and BG-11 media

Algae culture media are typically designed to provide nutritional requirements for both experimental studies and strain maintenance. These media are usually synthetic (chemically defined), predominantly made up of inorganic compounds, and can be prepared in liquid or solid form. Examples of culture media used for the maintenance and cultivation of *R. subcapitata* include those generally used for growing of freshwater green algae, such as Chu medium, Bold’s basal medium (BBM), or BG-11 medium (Watanabe 2005). Other culture media have been proposed and used by the researchers, but most of them only introduced minor changes, usually to reduce the number of stock solutions.

The Chu medium is composed by inorganic salts (calcium nitrate, dipotassium hydrogen phosphate,  magnesium sulfate, sodium carbonate, sodium metasilicate, and iron (III) chloride); lacks a chelator, vitamins, and trace metals (except Fe), and was designed to mimic lake water (Chu 1942). This medium is the base of different synthetic media developed for the growth of freshwater algae (Andersen et al. 2005).

BBM is derived from a modified version of Bristol’s solution (Bold 1949; Nichols and Bold 1965); this medium also lacks vitamins but presents trace metals (Zn, Mn, Mo, Cu, and Co), and a chelator (ethylenediaminetetraacetic acid, EDTA) (Andersen et al. 2005). A modification of Bold’s medium, known as modified Bolds 3N medium, presents an increased nitrate concentration and three vitamins (thiamine, biotin, and cyanocobalamin) (UTEX 2023b).

BG-11 medium is derived from medium No. 11 described by Hughes et al. (1958), for the culture of cyanobacteria, having increased the concentration of sodium nitrate and modified the trace elements solution (Allen 1968; Rippka et al. 1979).

### OECD and US-EPA medium

Probably, the most used culture media for the growth of *R. subcapitata* are OECD TG 201 medium (OECD 2011), in accordance with ISO 8692 (commonly referred to as OECD medium), and the US-EPA medium AAP, also according to ASTM (commonly referred to as US-EPA medium) (OECD 2011; US-EPA 2012), which are recommended by OECD and US-EPA, respectively. These two media present similar compositions in terms of the chemical elements, but diverge in their concentrations, the nitrogen source, and initial pH value.

The OECD medium presents as main constituents 7.1 mg L^−1^ C (50.0 mg L^−1^ sodium hydrogencarbonate, NaHCO_3_), 3.93 mg L^−1^ N (as NH_4_^+^), 0.115 mg L^−1^ Mn, and 0.100 mg L^−1^ of a chelating agent: disodium ethylenediaminetetraacetate dehydrate, Na_2_EDTA.2H_2_O. US-EPA medium is composed of similar concentrations of potassium (K), magnesium (Mg), and Mn, a higher concentration of N (4.20 mg L^−1^), as NO_3_^−^, Fe (0.0330 mg L^−1^ Fe), and chelating agent (0.300 mg L^−1^ Na_2_EDTA.2H_2_O) and lower content in C (2.14 mg L^−1^; 15.0 mg L^−1^ NaHCO_3_), P (0.186 mg L^−1^), K (0.469 mg L^−1^), sodium (Na) (11.0 mg L^−1^), and calcium (Ca) (1.20 mg L^−1^). Other differences between these two culture media, concerning P and trace elements (Cu and Zn) concentrations, were described above.

OECD and US-EPA media present pH values of 8.1 and 7.5, respectively. The pH of the culture medium is an important factor since it can affect the chemical speciation and, consequently, the bioavailability of the media components for algal nutrition as well as that of the toxicants, if this is the purpose of the study. For instance, a higher pH can originate chemical precipitation, in the form of hydroxides, of some micronutrients (metals). Although both media use NaHCO_3_ as a pH buffer, the OECD has a higher buffering capacity than the US-EPA medium, since it contains 50 mg L^−1^ NaHCO_3_, while US-EPA contains 15 mg L^−1^ NaHCO_3_ (OECD 2011).

The EDTA present in the culture medium promotes metal chelation, binding particularly divalent cations (Ca and Mg) which reduces their availability to the alga and avoids competition with essential micronutrients (Canuel et al. 2021). EDTA also has the function of preventing the Fe precipitation, present in the culture medium, and thus maintaining its bioavailability over the alga cultivation period (Pascual et al. 2020).

## Alga maintenance and preservation

The maintenance and storage of *R. subcapitata* play an important role in the preservation and dissemination of the alga for research and applied purposes. The strain, after being received from a commercial culture collection, is easily cultured using the aseptic technique and routinely maintained in the laboratory by sub-cultured, once a week, in an appropriate culture medium, such as OECD. By this process (serial transfer), the microalga can be maintained indefinitely. A small aliquot (5–10% of the culture volume) of a culture 4–8 days old is inoculated into a new flask containing a fresh culture medium and is incubated under optimal conditions. In many applications (such as toxicity assays), it is imperative to use, as inoculum, cells in the exponential phase of growth, which is obtained by incubating the cells for 2–3 days in a fresh culture medium (OECD 2011; US-EPA 2012). A recent study alerted to the risk that entails the long-term sub-culturing practice. The comparison of genome sequences among the same NIVA-CHL1 strain, available from eight (8) repositories, revealed the presence of mutations in non-coding and in coding regions (where some of them can affect protein function) in algae from some collections as well as different sensitiveness to 3,5-dichlorophenol and NaCl (Yamagishi et al. 2020).

For long-term maintenance of *R. subcapitata* (for several months), the strain is placed in a culture medium containing agar, in sterile Petri dishes or test tubes, incubated for approximately 1 week under optimal conditions, and then placed in the dark at 4 °C (OECD 2011; US-EPA 2012).

Preservation of the strain for a longer period of time can be achieved by freeze-drying or cryopreservation. In the last method, living microalgae are placed at a sufficiently low temperature that they are completely inactive metabolically; microalgae restore their normal physiological state after thawing (Brand et al. 2013). A cryoprotectant, such as dimethyl sulfoxide (DMSO), is added to the culture of *R. subcapitata*, to protect against cellular damage during cooling and during subsequent heating to revive the culture. Thus, the culture in a cryoprotective solution is gradually cooled to a very low temperature (ideally to less than − 50 °C) and, subsequently, stored at a temperature lower than − 130 °C (for instance, − 196 °C) in liquid nitrogen (Brand et al. 2013; Yamagishi et al. 2020). Cryopreservation of algal cells is considered the most reliable method for preserving microalgae vitality and genetic integrity over long periods of time (Brand et al. 2013; ATCC 2023). Many large culture collections, such as ATCC, CCAP, NIES, SAG, and UTEX, maintain both actively growing and cryopreserved cultures (ATCC 2023; CCAP 2023b; NIES 2023; SAG 2023a; UTEX 2023c).

## Factors affecting growth

### Light conditions

Light is one of the most important factors that affect the autotrophic growth of algae since it is the primary energy source for its metabolism. Light conditions, i.e., intensity, spectral composition, and duration of exposure, influence the physiological and biochemical processes of algae and, consequently, their growth and biomass composition (Maltsev et al. 2021). Therefore, the control of lighting conditions is of great importance, whether from a more fundamental (to obtain consistent algal growth profiles) or industrial point of view (use of photobioreactors for large-scale cultures); in the latter case, lighting corresponds to one of the main costs associated with indoor microalgae cultures (Gutierrez-Wing et al. 2012).

#### Intensity

At low light intensity, the photosynthetic rate of algae raises, almost linearly, with the increase of light intensity, until reaching a saturation point. A further increase does not raise the photosynthetic rate. Excessive light can cause photoinhibition and damage to the photosynthetic apparatus (Maltsev et al. 2021). Thus, below the saturation point, algal growth is limited by light, while above the saturation point, growth is inhibited by light (Lee et al. 2015). It was found that *R. subcapitata* cultivation under light-limited conditions increases algal phenotypic heterogeneity and can differentiate algal pigments distribution. On the contrary, without light limitation, algal cells tend to present a small range of phenotypic profiles, allowing a fast growth (Fontana et al. 2019). Optimal light intensity for microalgae growth is within a range of 26–400 µmol photons m^−2^ s^−1^ (Maltsev et al. 2021). US-EPA (2012) recommends a light intensity of 4300 lx (60 µmol photons m^−2^ s^−1^, considering a “cool-white” fluorescent lamp) for the cultivation of *R. subcapitata*, while OECD (2011) suggests a light intensity in a range of 4400–8880 lx (60–120 µmol photons m^−2^ s^−1^, with a “cool white” fluorescent lamp) to maximize algal growth. If algal cultivation takes place in the laboratory, to ensure maximum homogeneity of culture lighting, it is convenient that the flasks containing the culture are shaken and randomly changed in the incubator, in order that light intensity in the flasks should not vary more than ± 15% from the previously selected value (US-EPA 2012).

#### Spectral composition

Light quality, i.e., the spectral composition (which is related to the color or wavelength of the light), influences metabolic processes in microalgae and, consequently, their growth. The light flux of a specific spectral region can promote predominantly the accumulation of carbohydrates, lipids, proteins, or pigments in algal cells (Maltsev et al. 2021). *R. subcapitata* can use a broad spectrum of light (OECD 2011). Several studies have compared the effects of different light sources and colors on algal growth and pigment distribution/composition. Chlorophyll *a* (chl*a*) absorbs at the violet-blue spectrum area (390–450 nm) and at the orange-red zone (600–700 nm); chlorophyll *b* (chl*b*) absorbs at the blue-green spectrum area (390–500 nm) and yellow–red zone (600–680 nm) (Lichtenthaler and Welburn 1983). Thus, blue-white light (closer to daylight) should be more effective in promoting *R. subcapitata* photosynthesis and growth. This was confirmed by Patil et al. (2006) that compared the effect of three types of light: “interna”, “warm white”, and “cool white” on *R. subcapitata* growth. The combination of “cool white” (neutral to slightly blue light) with “interna light” (“warm white”, orange-to-yellow white light) resulted in maximum *R. subcapitata* growth; “warm white” showed the least growth. Gutierrez-Wing et al. (2012) also compared four light sources (metal halide, high-pressure sodium, Son Agro®, and cool white fluorescent lamp) for the growth, in a continuous regime, of *R. subcapitata*; the authors observed the lowest maximum growth rate with fluorescent light and the highest with Son Agro®. US-EPA (2012) recommends the use of “cool-white” (4000 K) fluorescent light and OECD (2011) advocates the illumination with “cool-white” or “daylight “ (6500 K) fluorescent lamps for the growth, in batch mode, of *R. subcapitata* to be subsequently used in toxicity bioassays. Illumination with “natural white” light-emitting diodes (LEDs) with a color temperature of 4000–4200 K is also adequate to promote the growth of *R. subcapitata* (Sousa et al. 2019).

#### Duration of lighting

The duration and frequency of light exposure also play an influence on algal growth and composition. The exposure of microalgae to continuous light (24 h light:0 h dark) generates enhanced photosynthetic performance and, as a consequence, improves microalgae growth. However, continuous lighting may induce photoinhibition processes, and in this case, the use of dark periods can be helpful in photosystems damage restoration and reduces energy consumption (Maltsev et al. 2021). US-EPA (2012) and OECD (2011) recommend the use of continuous illumination, for the propagation of *R. subcapitata* for toxicity tests. It was shown that continuous illumination increased the growth rate of *R. subcapitata* comparatively to 10:14 or 14:10 light to dark ratios (Gonçalves et al. 2014).

### Temperature

*R. subcapitata* proliferates, in a batch mode, within a wide range of temperatures, i.e., from 15 °C (or even less) to 37 °C (maximum growth temperature) (Reynolds et al. 1975). An increase in the specific growth rate of the alga was observed when the temperature raises from 15 to 21 °C (Pascual et al. 2022). An optimal growth temperature for *R. subcapitata* has been described in the range of 24 to 27 °C (Reynolds et al. 1975) or 22 to 30 °C (Fujimoto et al. 1994). OECD (2011) recommends the incubation of *R. subcapitata* in the range of 21 to 24 °C (± 2 °C) to obtain the microalgae to be used as inoculum in bioassays. US-EPA approves a temperature of 24 °C (± 2 °C) for algae growth for the same purpose (US-EPA 2012).

### pH

pH of the culture medium influences microalgae growth as it determines nutrients and CO_2_ bioavailability. During the growth, CO_2_ is absorbed by microalgae, as a consequence of photosynthesis, resulting in a gradual alkalinization of the medium (Wu et al. 2022). OECD (2011) recommends the evaluation of the pH of the culture medium, in the control assay, which should not increase by more than 1.5 units during the test period.

*R. subcapitata* growth is not adversely affected for initial culture medium pH values between 6 and 10 (US-EPA 1971). Within this pH range, the algal growth rate is generally higher, and biomass accumulates more rapidly. This range of pH is considered favorable for the algal physiological processes, such as nutrient uptake and photosynthesis (Chen and Durbin 1994).

### Salinity

Salinity, i.e., the concentration of salts dissolved in the water system, determines the distribution of microalgae in freshwater and marine ecosystems and has a great impact, particularly in coastal areas, converging the effect of run-off, rivers, and land. On the other hand, global warming, as a consequence of climate change, can cause changes in salinity patterns, which could have a strong impact on the distribution and composition of microalgae communities (Beardall and Raven 2004; Durack et al. 2012). In freshwater microalga, high salinity levels can disrupt the balance of osmotic pressure within the algal cells, affecting their ability to regulate water and nutrient uptake. This can lead to cellular dehydration, reduction of metabolic activity, and ultimately growth inhibition (Shetty et al. 2019). Salt (NaCl) stress can also lead to the overproduction of ROS that will perturb photosynthesis, and endanger microalgae survival in aquatic systems (Venâncio et al. 2017).

*R. subcapitata* seems to have a limited tolerance to the variation of salt concentration. In this regard, an inhibitory effect of NaCl over alga growth has been described with values of 72 h-EC_25_ of 2.96 g L^−1^ (Venâncio et al. 2017), 72 h-EC_50_ of 3.1 g L^−1^ (Sbrilli et al. 2003), and 96 h-EC_50_ of 4.1 g L^−1^ NaCl (Gonçalves et al. 2006); for comparative purposes, seawater present ~ 35 g L^−1^ of salt. Moreover, the exposure to low salinity (2.96 or 6.6 g L^−1^) along multi-generations led to a decrease in *R. subcapitata* growth rate with consequences to freshwater ecosystems and higher levels of the trophic chain (Venâncio et al. 2017). As a rule, US-EPA recommends that freshwater organisms only be used when salinity is less than 1 g L^−1^ (US-EPA 1991).

## Chronological aging (conditional senescence)

In unicellular microorganisms, aging can be described as replicative and chronological aging. Replicative aging corresponds to cellular deterioration due to the increase in the number of divisions. Chronological aging (also known as conditional senescence) is related to the degradation of the physiological state of cells, over time, as a result of the absence of nutrients or the presence of toxic metabolites, which prevent cell division (Florea 2017).

When cultivated in a batch mode, in OECD medium, *R. subcapitata* cell population increases exponentially during about 3 days after inoculation, with a generation (doubling) time of ~ 10.4 h (Machado and Soares 2022); a similar value (9.8–11.1 h) is reported by OECD (OECD 2011). At this stage, the cell population is homogeneous, composed of healthy “young” cells, which divide at the maximum rate and are characterized by having the maximum content of photosynthetic pigments and photosynthetic activity, storing starch as a reserve material and presenting, predominantly, a lunate morphology. Nutrient limitation causes the algal cell division to stop and the culture enters into the stationary phase on the 5th day of incubation. The incubation of algae for more seven (7) days, under these conditions, leads to their (chronological) aging. The “old” cells of *R. subcapitata* are characterized by a fading of their green color, reduction in the content of photosynthetic pigments and photosynthetic activity, chloroplast shrinkage, and accumulation of lipids, as reserve compounds (Machado and Soares 2022). Some of the modifications associated with chronological aging, namely alterations in the photosynthetic apparatus, reduction of chlorophyll content, and accumulation of lipids, were also observed in different algae, particularly in the genus *Chlamydomonas* (Damoo and Durnford 2021; Sato and Toyoshima 2021; Zamzam et al. 2022), and also in *Spongiochloris typica* (McLean 1968), *Phaeodactylum tricornutum*, and *Chaetoceros muelleri* (Liang et al. 2006). After the 12^th^ day of incubation, the cells of *R. subcapitata* begin to die (loss of cell membrane integrity) and the culture enters into the death phase. With advancing chronological aging, there is a marked loss of algal color, photosynthetic pigment content, and photosynthetic activity (Machado and Soares 2022).

The incubation of *R. subcapitata* in OECD medium, for 21 days, in a batch mode, originates alga cells with profound differences in their physiology and metabolism. These modifications are associated with algal growth and chronological aging, representing a switch (on the part of cells) from investment in reproduction (when nutrients are available) to a survival strategy when nutrients are scarce. This last condition (nutritional paucity) is frequently observed in nature and laboratorial conditions, when growth occurs in a batch regime (Gonidakis and Longo 2013). The characterization of the physiological and metabolic (“health”) status of *R. subcapitata* in these two scenarios is of great importance to allow reliable results in toxicity studies (where cells must be in exponential phase of growth) or the maximization of the production of high-value products, as detailed below (please see, section “Biotechnological applications”).

## Use of *R. subcapitata* in aquatic toxicology

**Fig. 4:**
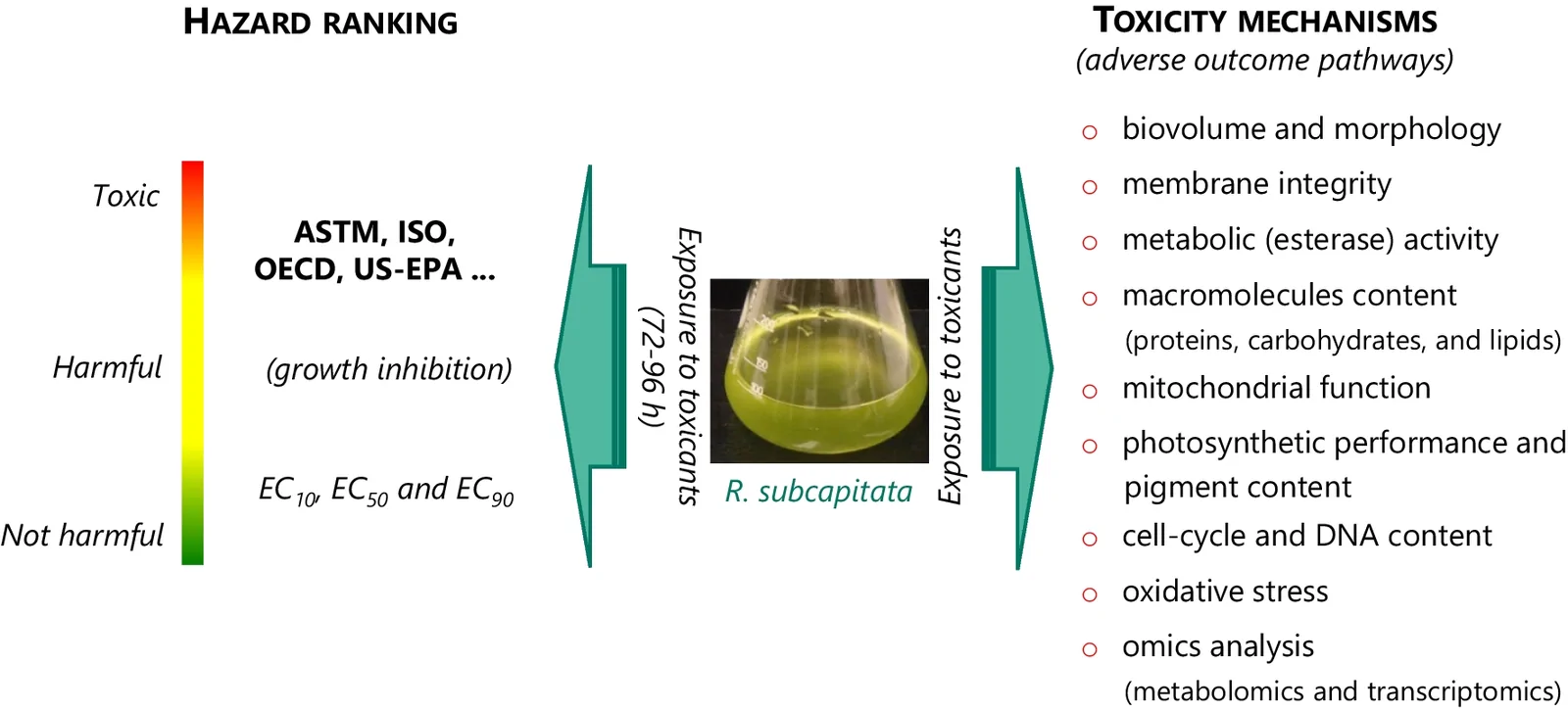
Use of *R. subcapitata* in aquatic toxicology. This alga has been used in hazard assessment of chemicals under different framework regulations, through standard international guidelines, which have as the endpoint the inhibition of algae growth. The use of different cellular and sub-cellular endpoints in *R. subcapitata* has allowed to establish adverse outcome pathways of different target chemical species

*R. subcapitata* has been mainly used in aquatic toxicology as a bioassay; more recently, this alga has also been used to elucidate the mode of action of toxics (Fig. 4), as detailed below.

### Ecotoxicity assessment of inorganic and organic toxics

#### Sensitivity of *R. subcapitata* to pollutants

*R. subcapitata* has a high sensitivity to inorganic chemical species (metals) and organic compounds (antibiotics, antiseptics, and herbicides) comparatively to other cell models such as fish (*Pimephales promelas*), crustaceans (*Thamnocephalus platyurus*), rotifer (*Brachionus calyciflorus*), protozoa (*Tetrahymena pyriformis*), and bacterium (*Photobacterium phosphoreum* and *Aliivibrio fischeri*) (George et al. 1995; Rojíčková-Padrtová et al. 1998) and even other microalgae such as *Scenedesmus quadricauda*, *Scenedesmus subspicatus*, *C. reinhardtii* (Wang et al. 2020), *Chlorella kessleri* (Rojickova-Padrtova and Marsalek 1999), and *Dunaliella tertiolecta* (Machado and Soares 2019b). Thus, a high sensitivity, in the order of µg L^−1^, of *R. subcapitata* to different chemical species has been described. Examples are (1) heavy metals, namely Cu, Ni, Zn, Cd, and Cr (Machado and Soares 2014; Al-Hasawi et al. 2020); (2) pharmaceuticals, as antibiotics (Fu et al. 2017; Machado and Soares 2019a; Li et al. 2021), antidepressants (Minguez et al. 2018), or non-steroidal anti-inflammatory drugs (Russo et al. 2023); (3) pesticides (Machado and Soares 2019b; Moreira et al. 2020); (4) biocides (Elersek et al. 2018; Machado and Soares 2021a); (5) nanoparticles (Lekamge et al. 2020); and (6) heterocyclic polyaromatic hydrocarbons (Çelik et al. 2023).

#### Experimental organism recommended by international organizations

Due to the high sensitivity of *R. subcapitata* to inorganic and organic toxics, ubiquitous distribution, ease of growing, short generation time, and low maintenance cost, this microorganism is widely used, as freshwater microalga, in the assessment of toxicity of effluents, receiving waters and aquatic pollutants (US-EPA 2002). For this purpose, standard guidelines from different international entities are available, such as ASTM, ISO, OECD, and US-EPA (Fig. 4); additionally, an extensive database of responses to this alga is available for a large number of organic and inorganic toxics—US-EPA ECOTOX Knowledgebase (US-EPA 2023a).

In the guidelines reported above for environmental hazard testing of chemicals in the framework of regulations, the test endpoint is the alga growth inhibition during the exposure period (72–96 h), expressed as % inhibition of specific growth rate or % inhibition of cell (or biomass) yield (cell concentration/biomass at the end of the exposure period minus the cell concentration/biomass at the beginning of the test). Although this endpoint (growth inhibition) is ecologically relevant, it only represents a response at the population level. Therefore, other endpoints have also been considered/proposed in the assessment of the toxic impact of chemicals, as well as in the elucidation of their mode of action (please, see below “Elucidation of mode of action of toxics”) and mechanistic effects on *R. subcapitata* at the sub-cellular level.

Considering that in nature toxics are not present single but in a complex matrix of chemical species that can interact with each other leading to synergistic, antagonistic, or and unpredictable effects, *R. subcapitata* has been used in the evaluation of mixtures (mainly binary) of toxics (Chèvre and Gregorio 2013). These studies started with the evaluation of pesticides (Mansano et al. 2017; Moreira et al. 2020) and have been extended to other classes of toxics, such as metals (Reis et al. 2021; Gebara et al. 2023), antibiotics (Chang et al. 2022), cytostatic drugs (Venâncio et al. 2023), biocides (Yang et al. 2008), and PAHs (Kreutzer et al. 2022).

### Elucidation of mode of action of toxics

#### Cellular and sub-cellular targets

Another aspect in which this microalga has been explored is in the elucidation of the mechanisms of pollutant toxicity. This issue is of particular interest since the elucidation (at the sub-cellular level) of the mode of action (MoA) of toxics, at environmentally relevant concentrations, using *R. subcapitata*, can establish adverse outcome pathways of targeted chemical species and help to adopt measures in the future to prevent their unwanted effects at higher trophic levels. For this purpose, different cellular and sub-cellular targets/endpoints have been considered (Fig. 4), such as (1) size/biovolume and shape (Machado and Soares 2014, 2020; Ostovich et al. 2023; McKeel et al. 2024); (2) membrane integrity (viability) (Nagai et al. 2011; Machado and Soares 2012a, 2015b); (3) metabolic (esterase) activity (Debenest et al. 2012; Machado and Soares 2013; Peng et al. 2019; Ciccia et al. 2023); (4) macromolecules content and distribution: proteins, carbohydrates, and lipids (Moreira et al. 2020; Ciccia et al. 2023; Ostovich et al. 2023); (5) photosynthetic performance and pigment content (chl*a*, chl*b*, and carotenoids) (Debenest et al. 2012; Peng et al. 2019; Almeida et al. 2021; dos Reis et al. 2022); (6) cell cycle and DNA content (Machado and Soares 2014; Sousa et al. 2018; Almeida et al 2019, 2021); (7) mitochondrial membrane potential (Machado and Soares 2015a; Almeida et al. 2019; Ciccia et al. 2023); (8) oxidative stress assessment: ROS production, enzymatic (superoxide dismutase and catalase activity) and non-enzymatic (reduced glutathione) defenses and lipid peroxidation (Machado and Soares 2012b, 2021b; Almeida et al. 2019; Filová et al. 2021); and (9) molecular (transcriptomic and metabolomics) analysis (Guo et al. 2021; Mizukami-Murata et al. 2021; Gómez-Martínez et al. 2023).

#### Examples of MoA of toxics clarified

As example of toxics whose MoA has been clarified using the microalga *R. subcapitata*, the following can be mentioned: (1) the antibiotics azithromycin (Almeida et al. 2021), clarithromycin (Peng et al. 2021), and erythromycin (Mo et al. 2023); (2) the herbicides MET (Machado and Soares 2021b) and diflufenican (Gómez-Martínez et al. 2023); (3) the heavy metals Cd, Cr, Cu, and Zn (Machado et al. 2015; Reis et al. 2021); (4) the antibacterial triclosan (Machado and Soares 2021a; Mo et al. 2022) and disinfection by-products (derived of haloacetic acids and halophenols) (Ciccia et al. 2023; Li et al. 2023); and (5) nanomaterials, such as metal(loid) oxide nanoparticles Al_2_O_3_, CuO, Mn_3_O_4_, NiO, SiO_2_, and SnO_2_ (Sousa et al. 2018, 2019), lithiated cobalt oxide nanosheets (Ostovich et al. 2023, 2024), micro/nano-plastics (polystyrene) (Reynolds et al. 2021), and carbon dots (McKeel et al. 2024).

For instance, azithromycin provokes in *R. subcapitata* a disturbance of the mechanism of energy dissipation of PSII centers, in the chloroplasts, leading to photo damaging; the interruption of the electron transport chain promotes over ROS generation, and consequently oxidative stress, with DNA and membranes injury (Almeida et al. 2021). MET, at environmentally relevant concentrations, induces a reduction of metabolic activity, photosynthetic efficiency, and electron transfer in the thylakoid membranes of chloroplasts in *R. subcapitata*. An increase in ROS accumulation and a decrease in enzymatic (superoxide dismutase and catalase) and non-enzymatic defenses (reduced glutathione levels) were also observed, leading to oxidative stress and consequent oxidative injury: lipid peroxidation and loss of cell membrane integrity (Machado and Soares 2021b). Probably, the deep disturbance of the physiology of the microalga leads to the impairment of its normal reproductive cycle and autospores release, translating into slowdown/arrest of the growth of *R. subcapitata*, increase in biovolume, alteration of cell shape, and formation of a palmelloid-like phenotype (Machado and Soares 2020, 2023).

## Biotechnological applications

**Fig. 5:**
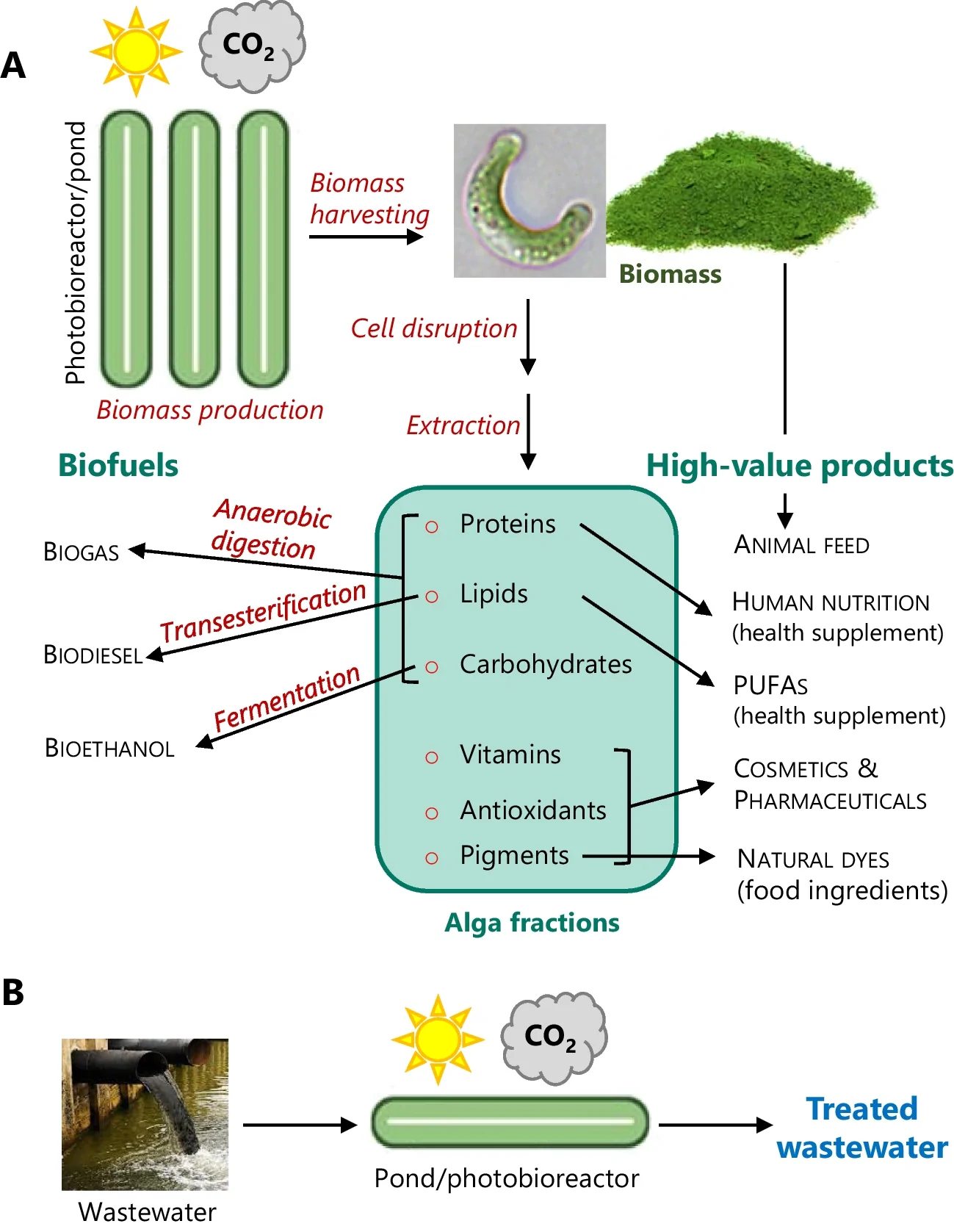
Potential biotechnological applications of *R. subcapitata*. A—production of biofuels (biogas, third-generation biodiesel, and bioethanol) and value-added products (such as essential fatty acids, proteins, vitamins, antioxidants, and pigments), which can be used in different sectors like animal feed, human nutrition, or pharmaceutical industry. B—wastewater bioremediation: removal of nutrients, inorganic species, polycyclic aromatic hydrocarbons, and pharmaceuticals of emerging concern from effluents

Microalgae can be used to mitigate environmental problems, such as the greenhouse effect (through the fixation of CO_2_ from the atmosphere) and the pollution caused by domestic and industrial effluents. Furthermore, due to its biochemical composition, microalgae can be a raw material, for the production of high-value products and biofuels, in a modern biorefinery context (Chew et al. 2017; Bhattacharya and Goswami 2020), as exemplified below, for the microalga *R. subcapitata*, and summarized in Fig. 5.

### Production of value-added biomolecules

#### Essential fatty acids

Microalgae are fast-growing unicellular organisms. In recent years, these microorganisms have been seen as an alternative source of functional food components, i.e., biomolecules that are believed to contribute to health and promote human well-being (Boukid and Castellari 2023). These molecules can be found either naturally in foods or intentionally added as ingredients, increasing their concentration (food fortification) or added as new constituents in the foods. Examples of functional food components are essential fatty acids, carotenoids, sterols, vitamins, and minerals (Shaikh 2022).

A typical example of functional food components are ω-3 fatty acids, due to their potential application in the prevention of cardiovascular diseases (Kaur et al. 2014), which can be used to fortify dairy products such as milk and yoghurt (Ganesan et al. 2014). Fatty acids that contain more than one double bond in their structure are collectively called polyunsaturated fatty acids (PUFAs) (Smith 2000). Among them, it is of special interest ω-3 fatty acids, such as *α*-linolenic acid (ALA), eicosapentaenoic acid (EPA), and docosahexaenoic acid (DHA). Humans are not able to synthetize ALA; EPA and DHA are produced using ALA as a precursor (Das 2006). Therefore, all three PUFAs are necessarily obtained by a nutritional route, such as oils from plant and animal sources (ALA) or from marine food sources, namely fish, fish oils, and algae (EPA and DHA). These lipids, coming from microalgae, can also be used as supplements for vegetarian and vegan diets, which often lack ω-3 fatty acids (Ganesan et al. 2014). It is in this context that *R. subcapitata* can be used as a source of ω-3 and ω-6 fatty acids (Patil et al. 2007). Recent work described the extraction of 62.1 mg of PUFA/g of *R. subcapitata*, wherein 43.6 mg/g of linolenic acid (Saliu et al. 2021). Besides the food industry, ω-3 and ω-6 fatty acids (such as linoleic acid; another essential fatty acid), produced by microalgae, can be important in the pharmaceutical industry as these compounds are precursors of prostaglandins and thromboxanes (Das 2006).

#### Pigments

Carotenoids are another functional food component available from algae. These compounds are consumed as dietary supplements, due to their antioxidant properties. The protective effect of carotenoids against serious disorders, such as heart and degenerative eye disease has been described (Shaikh 2022). The microalga *R. subcapitata* presents carotenoids concentration of 0.26 mg l^–1^ (Nascimento et al. 2020). Carotenoids produced by microalgae can also be used as dyes, being added as food ingredients (in food and beverages) as well as pigments in poultry and fish farming (Shaikh 2022).

Other pigments of potential commercial interest, associated with microalgae biomass, are chlorophylls. It is described, although there is not much scientific evidence to prove it, that chlorophylls have benefits for human health due to their antioxidant and anticancer properties (Pérez-Gálvez et al. 2020). *R. subcapitata* can accumulate about 3.6 mg l^–1^ chl*a* and 1.4 mg l^–1^ chl*b* (Nascimento et al. 2020), which opens up the possibility of its use as a source of chlorophyll (supplement), in the form of dried biomass, in the preparation of new food-enriched formulations, similar to what has been proposed for other microalgae; examples include cereal-based products, such as wheat crackers (Batista et al. 2019).

#### Proteins

In recent decades, there has been an increase in meat consumption, being foreseen an increase of 14% in the present decade (OECD-FAO 2023). Faced with this growing need for protein, microalgae emerge as an excellent alternative source of protein, containing essential amino acids (Lum et al. 2013). *R. subcapitata* contains about 42% of protein (Nascimento et al. 2020), and can be used both in human nutrition and in animal feed, namely for aquaculture (Patil et al. 2007; Nagappan et al. 2021). As described for other microalgae, *R. subcapitata* proteins can present other applications such as enzymes (Moreno-Garcia et al. 2017).

### Production of biofuels

#### Third-generation biodiesel

Replacing fossil fuels with biofuels is positively seen since it can reduce some unwanted aspects associated with fossil fuel production and use, such as the greenhouse gas emissions to the atmosphere (US-EPA 2023b). Due to the fact that diesel is an important fuel in the transport sector, there has been a growing interest in its production since it is a renewable, environmental friendly, and less toxic source of energy, compared to conventional diesel of fossil origin (Živković and Veljković 2018). Biodiesel can be produced from a wide variety of feedstocks, such as edible oilseed crops (first generation), vegetable oils from non-edible plants, animal fats, and waste cooking oils (second generation). Microalgae have been recognized as a potentially sustainable source for the production of third-generation biodiesel (microbiodiesel) because of their relatively high oil content, rapid biomass production, not requiring fertile land or food crops, and promotes CO_2_ sequestration (Pinzi and Dorado 2012).

In the case of microalgae, an accumulation of high lipid levels has been described, which may exceed 60% of the dry biomass, although it is more common for them to reach values of 20–50% (Pinzi and Dorado 2012). *R. subcapitata* presents a lipid content of ~ 27% (dry microalgae biomass), which is higher than that found in the microalga *Scenedesmus spinosus* and *Scenedesmus acuminatus*, thus presenting great potential for biofuels production (Nascimento et al. 2020). Usually, the increase in lipid levels occurs when microalgae are in the stationary phase of growth, upon nutrient limitation or under different stresses, such as temperature and light (Pinzi and Dorado 2012; Moreira et al. 2022). An increase in fatty acid content in *R. subcapitata* when cultivated under phosphate limitation has been described (Benasla and Hausler 2020), being able to reach a lipid content of ~ 32% (dry biomass) under nitrate limitation (Del Río et al. 2015).

Besides lipid content, other criteria should be considered in the selection of microalgae strains for microbiodiesel production, namely oil yield (productivity) and fatty acid composition (Nascimento et al. 2013; Del Río et al. 2015). The length of the carbon chain and the level of unsaturation of fatty acids have a direct impact on biodiesel properties, such as cetane number (a measure of fuel ignition properties), cold-flow characteristics, viscosity, and oxidative stability (Stansell et al. 2012). A screening of 12 strains (nine of *Chlorophyceae* and three of *Trebouxiophyceae*), showed that *R. subcapitata* grown in a batch regime presented a volumetric lipid productivity of 23 mg L^−1^ day^−1^ (Nascimento et al. 2013). Another study, conducted with 10 strains belonging to different genera, showed the great potential of *R. subcapitata* when grown in continuous culture (Del Río et al. 2015). The same authors showed that this alga presented a fatty acid productivity of 160 mg L^−1^ day^−1^, corresponding to 180 mg lipids L^−1^ day^−1^, when grown in continuous culture with 3–5 mmol L^−1^ nitrate; such productivity is clearly higher than the obtained with the oleaginous alga *Chlorococcum oleofaciens* and to those described in the literature for microalgae when grown continuously (Del Río et al. 2017). In addition, *R. subcapitata* produces a suitable fatty acid profile (originating a raw material enriched in saturated and monounsaturated fatty acids); under nitrogen-limited conditions, the % of C_16_:0 plus C_18_:1 fatty acids accounted for 74% of total fatty acids produced by the alga, with a linolenic acid content below 10%, which highlights their adequacy as an appropriate source of fatty acids for biodiesel production (Del Río et al. 2015, 2017). Priyanka et al. (2020) evaluated lipid production by *R. subcapitata* grown in simulated wastewater and, simultaneously, adopted a strategy in which fatty acids were bioconverted into the corresponding methyl esters (biodiesel feedstock) by in situ transesterification. The growth of *R. subcapitata*, immobilized in sodium alginate beads, for biofuel production, was also described (Benasla and Hausler 2018; 2020).

#### Biogas and bioethanol

The analysis of the composition of the dry microalga biomass of *R. subcapitata* revealed that it consists of approximately 25% of carbohydrates (Nascimento et al. 2020) and thus can be used as a raw material for biogas and bioethanol production. Biogas consists of a mixture containing mainly methane and carbon dioxide. A recent work showed the production of methane by anaerobic digestion of *R. subcapitata* biomass (used as co-substrate with olive mill solid waste) cultivated in olive oil industry effluents (Fernández-Rodríguez et al. 2022). On the other hand, bioethanol can be obtained from the whole microalgae biomass or the lipid-free portion. After pretreatment, usually a chemical and/or enzymatic hydrolysis, the free sugars (obtained from the biomass) are fermented to produce bioethanol (Wang et al. 2022).

### Bioremediation

The use of microalgae in bioremediation processes, namely in wastewater treatment, nutrient removal and recovery, and in the remediation of heavy metals and organic compounds has been described (Deviram et al. 2020).

#### Nutrient removal from wastewater

The treatment of olive oil industry effluents by *R. subcapitata* was described, which resulted in the removal of phosphate, nitrate, sugars, and soluble chemical oxygen demand. This methodology combined the mitigation of the environmental impact of effluents from the olive oil industry with the valorization of the by-products produced by this industry, in the context of a circular economy (Fernández-Rodríguez et al. 2022). Another approach joined the wastewater bioremediation (removal of phosphorus, nitrogen, and carbon organic matter) with bioelectricity generation through the use of a microalgae-microbial fuel cell, containing *Escherichia coli* in the anodic chamber and *R. subcapitata* in the cathodic chamber (Ribeiro et al. 2022).

#### Removal of inorganic species

The bioremediation of inorganic chemical species by *R. subcapitata* has been described. Thus, Pitre et al. (2014), simulating the conditions found in the effluents of the aluminum industry, showed that *R. subcapitata* was particularly appropriate (compared to other microalgae, such as *C. reinhardtii*, *C. vulgaris*, and *Scenedesmus obliquus*) to remove aluminum and fluoride. Other studies also evidenced the suitability of *R. subcapitata* in the bioremediation of inorganic ceramic nanomaterials (Jakubczak et al. 2022) or to remove copper and lead from industrial effluents from battery manufacture (Cygnarowska 2023).

#### Bioremediation of polycyclic aromatic hydrocarbons, and pharmaceuticals of emerging concern

PAHs are commonly found in petroleum-contaminated waters. *R. subcapitata* seems to be particularly suited to treat this type of pollutant, since it proved to be more effective than other microalgae (such as *C. vulgaris*, *Scenedesmus platydiscus*, and *S. quadricauda*), removing almost completely and degrade, individually or in a mixture, different PAHs, such as phenanthrene, fluoranthene, pyrene (Chan et al. 2006; Lei et al. 2007) and different kinds of methylphenanthrenes (Luo et al. 2020).

The presence of pharmaceutical compounds in the aquatic systems has raised in recent years as a consequence of the increased consumption of both humans and veterinary. Since conventional wastewater treatment plants are not completely efficient in the removal of these compounds, new treatment strategies have been proposed including biological processes (Patel et al. 2019). Thus, the use of *R. subcapitata* in the removal of nine (9) antibiotics and one (1) antidepressant was evaluated. A removal between 10 and 77%, according to the antibiotic, was observed while the removal of the antidepressant was only 11% (Hom-Diaz et al. 2022). This alga also efficiently (88–100%) removes and biodegrades the steroid hormones β-estradiol and 17α-ethinylestradiol (Della Greca et al. 2008; Hom-Diaz et al. 2015; Wang et al. 2023).

## Final remarks

Despite its notoriety, which comes mainly from the fact that it is recommended by several international organizations, as a sensitive and reliable indicator of ecotoxicity, the microalga *R. subcapitata* remains, in many aspects, an “illustrious” unknown. The number of works published in the last 20 years with the *R. subcapitata* is relatively modest (5.7 times lesser) when compared, for example, with the green alga *C. reinhardtii*, as can be seen in Supplementary Material (Fig. S1).

Studies of a more fundamental character, for example on its ultra-structure, and metabolism are scarce and, normally, with several years old, which demonstrates the less attention that this alga has received from the scientific community, in this type of studies. In fact, *R. subcapitata* has been used, mainly, in an applied perspective, in environmental studies, as is confirmed in a search in Web of Science-Clarivate Analytics, using as a topic “*Raphidocelis subcapitata* or *Pseudokirchneriella subcapitata* or *Selenastrum capricornutum*”, “all fields”, from 2005 to 2024 (search: 10 January 2024). About 63% and 28% of the papers published in the last 20 years, with this alga, were in the categories of Web of Science “Environmental Sciences” and “Toxicology”, respectively, while only ~ 3% were published in the category “Biochemistry and Molecular Biology”. The same can be said with regard to the use of this alga in Biotechnology, where only ~ 5% of published papers are in the field of “Biotechnology and Applied Microbiology”.

However, as was shown above, the works published in recent years are very encouraging in terms of the possibilities of *R. subcapitata* being at the origin of the production of value-added biomolecules and biofuels or being associated with bioremediation processes. As a consequence of the composition of its biomass (which contains, among other molecules, proteins, lipids, carbohydrates, vitamins, and photosynthetic pigments), *R. subcapitata* has a high potential, as a raw material, for obtaining biofuels and high-value products, in a biorefinery context. For instance, lipids are suited for third-generation biodiesel (microbiodiesel) production, while residual biomass, rich in carbohydrates, is appropriate for bioethanol and biogas production. Other application of this microalgae includes the production of natural dyes, pharmaceutical compounds and nutrients for animal feed, and innovative functional food products, containing healthy beneficial compounds for human nutrition, as it was depicted in Fig. 5. Since the biotechnological potential of *R. subcapitata* has so far been little explored, there is still a long and promising way to go in this field.

## Supplementary Information

Below is the link to the electronic supplementary material.

Supplementary file1 (PDF 116 KB)

## Declarations

This article does not contain any studies with human participants or animals performed by the authors.

## References

[CR1] Al-Hasawi ZM, Abdel-Hamid MI, Almutairi AW, Touliabah HE (2020) Response of *Pseudokirchneriella subcapitata* in free and alginate immobilized cells to heavy metals toxicity. Molecules 25:2847. 10.3390/molecules25122847 32575616 10.3390/molecules25122847 PMC7355555

[CR2] Allen MM (1968) Simple conditions for growth of unicellular blue-green algae on plates. J Phycol 4:1–4. 10.1111/j.1529-8817.1968.tb04667.x 27067764 10.1111/j.1529-8817.1968.tb04667.x

[CR3] Almeida AC, Gomes T, Habuda-Stanić M, Lomba JAB, Romić Ž, Turkalj JV, Lillicrap A (2019) Characterization of multiple biomarker responses using flow cytometry to improve environmental hazard assessment with the green microalgae *Raphidocelis subcapitata*. Sci Total Environ 687:827–838. 10.1016/j.scitotenv.2019.06.124 31412486 10.1016/j.scitotenv.2019.06.124

[CR4] Almeida AC, Gomes T, Lomba JAB, Lillicrap A (2021) Specific toxicity of azithromycin to the freshwater microalga *Raphidocelis subcapitata*. Ecotoxicol Environ Saf 222:112553. 10.1016/j.ecoenv.2021.112553 34325198 10.1016/j.ecoenv.2021.112553

[CR5] ANACC (2023) *Pseudokirchneriella subcapitata*. Strain no. CS-327. Australian National Algae Culture Collection. https://www.csiro.au/en/about/facilities-collections/Collections/ANACC. Accessed 10 Jul 2023

[CR6] Andersen RA, Berges JA, Harrison PJH, Watanabe MM (2005) Recipes for freshwater and seawater media (Appendix A). In: Andersen RA (ed) Algal culturing techniques. Elsevier Academic Press, New York, pp 429–538

[CR7] Arbildua JJ, Villavicencio G, Urrestarazu P, Opazo M, Brix KV, Adams WJ, Rodriguez PH (2017) Effect of Fe (III) on *Pseudokirchneriella subcapitata* at circumneutral pH in standard laboratory tests is explained by nutrient sequestration. Environ Toxicol Chem 36:952–958. 10.1002/etc.3609 27591735 10.1002/etc.3609

[CR8] ASTM (2021) Standard guide for conducting static toxicity tests with microalgae. (ASTM E1218–21). American Society for Testing and Materials. https://www.astm.org/e1218-21.html

[CR9] ATCC (2023) Pseudokirchneriella* subcapitata* (Korshikov) Hindak. Strain no. 22662. American Type Culture Collection. https://www.atcc.org/products/22662. Accessed 10 Jul 2023

[CR10] Barinova S, Bragina T, Nevo E (2009) Algal species diversity of arid region lakes in Kazakhstan and Israel. Community Ecol 10:7–16. 10.1556/comec.10.2009.1.2

[CR11] Batista AP, Niccolai A, Bursic I, Sousa I, Raymundo A, Rodolfi L, Biondi N, Tredici MR (2019) Microalgae as functional ingredients in savory food products: application to wheat crackers. Foods 8:611. 10.3390/foods8120611 31771197 10.3390/foods8120611 PMC6963871

[CR12] Beardall J, Raven JA (2004) The potential effects of global climate change on microalgal photosynthesis, growth and ecology. Phycologia 43:26–40. 10.2216/i0031-8884-43-1-26.1

[CR13] Benasla A, Hausler R (2018) Optimisation of growth of *Raphidocelis subcapitata* immobilised for biofuel production: influence of alginate and CaCl_2_ concentrations on growth. Environments 5:60. 10.3390/environments5050060

[CR14] Benasla A, Hausler R (2020) Growth and production of lipids in *Raphidocelis subcapitata* immobilized in sodium alginate beads. Energies 13:506. 10.3390/en13020506

[CR15] Bhattacharya M, Goswami S (2020) Microalgae – a green multi-product biorefinery for future industrial prospects. Biocatal Agric Biotechnol 25:101580. 10.1016/j.bcab.2020.101580

[CR16] Blaise C, Vasseur P (2005) Microplate toxicity test. In: Blaise C, Férard JF (eds) Small-scale freshwater toxicity investigations, vol 1. Springer. Dordrecht, Netherlands, pp 137–179

[CR17] Bold HC (1949) The morphology of *Chlamydomonas chlamydogama* sp. nov. Bull Torrey Bot Club 76:101–108. 10.2307/2482218

[CR18] Borges VRP (2016) A computer-assisted approach to supporting taxonomical classification of freshwater green microalga images. PhD thesis in Computer Science and Computational Mathematics. São Paulo University, São Carlos, Brazil

[CR19] Borowitzka MA (2018) The ‘stress’ concept in microalgal biology—homeostasis, acclimation and adaptation. J Appl Phycol 30:2815–2825. 10.1007/s10811-018-1399-0

[CR20] Borowitzka MA, Siva CJ (2007) The taxonomy of the genus *Dunaliella* (*Chlorophyta, Dunaliellales*) with emphasis on the marine and halophilic species. J Appl Phycol 19:567–590. 10.1007/s10811-007-9171-x

[CR21] Boukid F, Castellari M (2023) Algae as nutritional and functional food sources. Foods 12:122. 10.3390/foods12010122 10.3390/foods12010122 PMC9818788 36613337

[CR22] Brand JJ, Andersen RA, Nobles DR Jr (2013) Maintenance of microalgae in culture collections. In: Richmond A, Hu Q (eds) Handbook of microalgal culture: applied phycology and biotechnology, 2nd edn. John Wiley & Sons Ltd, West Sussex, UK, pp 80–89

[CR23] Cambra-Sánchez J, Alvarez-Cobelas M, Aboal M (1998) Lista florística y bibliográfica de los clorófitos (*Chlorophyta*) de la Península Ibérica. Islas Baleares e Islas Canarias Asociación Española de Limnología, Burgos

[CR24] Canuel E, Vaz C, Matias WG, Dewez D (2021) Interaction effect of EDTA, salinity, and oxide nanoparticles on alga *Chlamydomonas reinhardtii* and *Chlamydomonas euryale*. Plants 10:2118. 10.3390/plants10102118 34685927 10.3390/plants10102118 PMC8541132

[CR25] Cărăus I (2002) Algae of Romania. A distributional checklist of actual algae. Stud Si Cercet Biol Univ Bacau 7:1–809

[CR26] CCALA (2023) *Raphidocelis subcapitata*. Strain 433. Culture Collection of Autotrophic Organisms . ) https://ccala.butbn.cas.cz/en/raphidocelis-subcapitata-korshikov-nygaard-et-al. Accessed 7 Jul 2023

[CR27] CCAP (2023a) Culture supply. Culture Collection of Algae & Protozoa. https://www.ccap.ac.uk/index.php/our-services/how-to-order/ Accessed 10 Jul 2023

[CR28] CCAP (2023b) *Raphidocelis *subcapitata. Strain no. 278/4. Culture Collection of Algae and Protozoa. https://www.ccap.ac.uk/catalogue/strain-278-4. Accessed 10 Jul 2023

[CR29] Çelik G, Stolte S, Markiewicz M (2023) NSO-heterocyclic PAHs – controlled exposure study reveals high acute aquatic toxicity. J Hazard Mater 460:132428. 10.1016/j.jhazmat.2023.132428 37690200 10.1016/j.jhazmat.2023.132428

[CR30] Chan SMN, Luan T, Wong MH, Tam NFY (2006) Removal and biodegradation of polycyclic aromatic hydrocarbons by *Selenastrum capricornutum*. Environ Toxicol Chem 25:1772–1779. 10.1897/05-354r.1 16833137 10.1897/05-354r.1

[CR31] Chang F, Yi M, Li H, Wang J, Zhao X, Hu X, Qi Q (2022) Antibiotic toxicity isolated and as binary mixture to freshwater algae *Raphidocelis subcapitata*: growth inhibition, prediction model, and environmental risk assessment. Toxics 10:739. 10.3390/toxics10120739 36548572 10.3390/toxics10120739 PMC9785756

[CR32] Chen C, Durbin E (1994) Effects of pH on the growth and carbon uptake of marine phytoplankton. Mar Ecol Prog Ser 109:83–94. 10.3354/meps109083

[CR33] Chèvre N, Gregorio V (2013) Mixture effects in ecotoxicology. In: Férard J-F, Blaise C (eds) Encyclopedia of aquatic ecotoxicology. Springer, Netherlands, Dordrecht, pp 729–736

[CR34] Chew KW, Yap JY, Show PL, Suan NH, Juan JC, Ling TC, Lee D-J, Chang J-S (2017) Microalgae biorefinery: high value products perspectives. Bioresour Technol 229:53–62. 10.1016/j.biortech.2017.01.006 28107722 10.1016/j.biortech.2017.01.006

[CR35] Chu SP (1942) The influence of the mineral composition of the medium on the growth of planktonic algae: Part I. Methods and Culture Media J Ecol 30:284–325. 10.2307/2256574

[CR36] Ciccia T, Pandard P, Ciffroy P, Urien N, Lafay L, Bado-Nilles A (2023) Sub-lethal toxicity of five disinfection by-products on microalgae determined by flow cytometry – lines of evidence for adverse outcome pathways. Ecotoxicol Environ Saf 266:115582. 10.1016/j.ecoenv.2023.115582 37862747 10.1016/j.ecoenv.2023.115582

[CR37] CPCC (2023) *Raphidocelis *subcapitata. Strain no. 37. Canadian Phycological Culture Centre. https://uwaterloo.ca/canadian-phycological-culture-centre/services-provided. Accessed 10 Jul 2023

[CR38] Cygnarowska K (2023) The use of algae to remove copper and lead from industrial wastewater. Geol Geophys Environ 49:85–93. 10.7494/geol.2023.49.1.85

[CR39] Damoo DY, Durnford DG (2021) Long-term survival of *Chlamydomonas reinhardtii* during conditional senescence. Arch Microbiol 203:5333–5344. 10.1007/s00203-021-02508-y 34383108 10.1007/s00203-021-02508-y

[CR40] Das UN (2006) Essential fatty acids: biochemistry, physiology and pathology. Biotechnol J 1:420–439. 10.1002/biot.200600012 16892270 10.1002/biot.200600012

[CR41] de Carpentier F, Lemaire S, Danon A (2019) When unity is strength: the strategies used by *Chlamydomonas* to survive environmental stresses. Cells 8:1307. 10.3390/cells8111307 31652831 10.3390/cells8111307 PMC6912462

[CR42] Debenest T, Turcotte P, Gagné F, Gagnon C, Blaise C (2012) Ecotoxicological impacts of effluents generated by oil sands bitumen extraction and oil sands lixiviation on *Pseudokirchneriella subcapitata*. Aquat Toxicol 112–113:83–91. 10.1016/j.aquatox.2012.01.021 22387878 10.1016/j.aquatox.2012.01.021

[CR43] Del Río E, Armendáriz A, García-Gómez E, García-González M, Guerrero MG (2015) Continuous culture methodology for the screening of microalgae for oil. J Biotechnol 195:103–107. 10.1016/j.jbiotec.2014.12.024 25562422 10.1016/j.jbiotec.2014.12.024

[CR44] Del Río E, García-Gómez E, Moreno J, G. Guerrero M, García-González M, (2017) Microalgae for oil. Assessment of fatty acid productivity in continuous culture by two high-yield strains, *Chlorococcum oleofaciens* and *Pseudokirchneriella subcapitata*. Algal Res 23:37–42. 10.1016/j.algal.2017.01.003

[CR45] Della Greca M, Pinto G, Pistillo P, Pollio A, Previtera L, Temussi F (2008) Biotransformation of ethinylestradiol by microalgae. Chemosphere 70:2047–2053. 10.1016/j.chemosphere.2007.09.011 17950412 10.1016/j.chemosphere.2007.09.011

[CR46] Deviram G, Mathimani T, Anto S, Ahamed TS, Ananth DA, Pugazhendhi A (2020) Applications of microalgal and cyanobacterial biomass on a way to safe, cleaner and a sustainable environment. J Clean Prod 253:119770. 10.1016/j.jclepro.2019.119770

[CR47] dos Reis LL, de Alho L, OG, Abreu CB de, Melão M da GG, (2021) Using multiple endpoints to assess the toxicity of cadmium and cobalt for chlorophycean *Raphidocelis subcapitata*. Ecotoxicol Environ Saf 208:111628. 10.1016/j.ecoenv.2020.111628 33396148 10.1016/j.ecoenv.2020.111628

[CR48] dos Reis LL, de Oliveira Lays, Alho Gonçalves, de Abreu CB, Gebara RC, da Silva Adrislaine, Mansano Maria, da Graça Gama Melão, (2022) Effects of cadmium and cobalt mixtures on growth and photosynthesis of *Raphidocelis subcapitata* (*Chlorophyceae*). Aquat Toxicol 244:106077. 10.1016/j.aquatox.2022.106077 35091369 10.1016/j.aquatox.2022.106077

[CR49] Durack PJ, Wijffels SE, Matear RJ (2012) Ocean salinities reveal strong global water cycle intensification during 1950 to 2000. Science 336:455–458. 10.1126/science.1212222 22539717 10.1126/science.1212222

[CR50] El-Agawany NI, Kaamoush MIA (2023) Role of zinc as an essential microelement for algal growth and concerns about its potential environmental risks. Environ Sci Pollut Res 30:71900–71911. 10.1007/s11356-022-20536-z 10.1007/s11356-022-20536-z PMC10257637 35551598

[CR51] Elersek T, Ženko M, Filipič M (2018) Ecotoxicity of disinfectant benzalkonium chloride and its mixture with antineoplastic drug 5-fluorouracil towards alga *Pseudokirchneriella subcapitata*. PeerJ 6:e4986. 10.7717/peerj.4986 29938131 10.7717/peerj.4986 PMC6011824

[CR52] Fawley MW, Dean ML, Dimmer SK, Fawley KP (2006) Evaluating the morphospecies concept in the *Selenastraceae* (*Chlorophyceae, Chlorophyta*). J Phycol 42:142–154. 10.1111/j.1529-8817.2006.00169.x

[CR53] Fernández-Rodríguez MJ, de la Lama-Calvente D, García-González M, Moreno-Fernández J, Jiménez-Rodríguez A, Borja R, Rincón-Llorente B (2022) Integral valorization of two-phase olive mill solid waste (OMSW) and related washing waters by anaerobic co-digestion of OMSW and the microalga *Raphidocelis subcapitata* cultivated in these effluents. J Agric Food Chem 70:3219–3227. 10.1021/acs.jafc.1c08100 35254817 10.1021/acs.jafc.1c08100 PMC8931757

[CR54] Filová A, Fargašová A, Molnárová M (2021) Cu, Ni, and Zn effects on basic physiological and stress parameters of *Raphidocelis subcapitata* algae. Environ Sci Pollut Res Int 28:58426–58441. 10.1007/s11356-021-14778-6 34115300 10.1007/s11356-021-14778-6

[CR55] Fisher RM, Bell T, West SA (2016) Multicellular group formation in response to predators in the alga *Chlorella vulgaris*. J Evol Biol 29:551–559. 10.1111/jeb.12804 26663204 10.1111/jeb.12804

[CR56] Florea M (2017) Aging and immortality in unicellular species. Mech Ageing Dev 167:5–15. 10.1016/j.mad.2017.08.006 28844968 10.1016/j.mad.2017.08.006

[CR57] Fontana S, Thomas MK, Reyes M, Pomati F (2019) Light limitation increases multidimensional trait evenness in phytoplankton populations. ISME J 13:1159–1167. 10.1038/s41396-018-0320-9 30617295 10.1038/s41396-018-0320-9 PMC6474219

[CR58] Franklin NM, Stauber JL, Lim RP (2001) Development of flow cytometry-based algal bioassays for assessing toxicity of copper in natural waters. Environ Toxicol Chem 20:160–170. https://doi.org/10.1002/etc.5620200118 11351404

[CR59] Fu L, Huang T, Wang S, Wang X, Su L, Li C, Zhao Y (2017) Toxicity of 13 different antibiotics towards freshwater green algae *Pseudokirchneriella subcapitata* and their modes of action. Chemosphere 168:217–222. 10.1016/j.chemosphere.2016.10.043 27783962 10.1016/j.chemosphere.2016.10.043

[CR60] Fučíková K, Leliaert F, Cooper ED, Škaloud P, D’Hondt S, De Clerck O, Gurgel CFD, Lewis LA, Lewis PO, Lopez-Bautista JM, Delwiche CF, Verbruggen H (2014a) New phylogenetic hypotheses for the core *Chlorophyta* based on chloroplast sequence data. Front Ecol Evol 2:63. 10.3389/fevo.2014.00063

[CR61] Fučíková K, Lewis PO, Lewis LA (2014b) Putting incertae sedis taxa in their place: a proposal for ten new families and three new genera in *Sphaeropleales* (*Chlorophyceae, Chlorophyta*). J Phycol 50:14–25. 10.1111/jpy.12118 26988005 10.1111/jpy.12118

[CR62] Fujimoto N, Inamori Y, Sugiura N, Sudo R (1994) Effects of temperature change on algal growth. Environ Technol 15:497–500. 10.1080/09593339409385455

[CR63] Ganesan B, Brothersen C, McMahon DJ (2014) Fortification of foods with omega-3 polyunsaturated fatty acids. Crit Rev Food Sci Nutr 54:98–114. 10.1080/10408398.2011.578221 24188235 10.1080/10408398.2011.578221

[CR64] Gebara RC, de Oliveira Lays, Alho Gonçalves, da Silva Adrislaine, Mansano Giseli Swerts, Rocha Maria, da Graça Gama Melão, (2023) Single and combined effects of Zn and Al on photosystem II of the green microalgae *Raphidocelis subcapitata* assessed by pulse-amplitude modulated (PAM) fluorometry. Aquat Toxicol 254:106369. 10.1016/j.aquatox.2022.106369 36502662 10.1016/j.aquatox.2022.106369

[CR65] George DB, Berk SG, Adams VD, Ting RS, Roberts RO, Parks LH, Lott RC (1995) Toxicity of alum sludge extracts to a freshwater alga, protozoan, fish, and marine bacterium. Arch Environ Contam Toxicol 29:149–158. 10.1007/BF00212964

[CR66] Giordano M, Beardall J, Raven JA (2005) CO_2_ concentrating mechanisms in algae: mechanisms, environmental modulation, and evolution. Annu Rev Plant Biol 56:99–131. 10.1146/annurev.arplant.56.032604.144052 15862091 10.1146/annurev.arplant.56.032604.144052

[CR67] Gómez-Martínez D, Bengtson J, Nilsson AK, Clarke AK, Nilsson RH, Kristiansson E, Corcoll N (2023) Phenotypic and transcriptomic acclimation of the green microalga *Raphidocelis subcapitata* to high environmental levels of the herbicide diflufenican. Sci Total Environ 875:162604. 10.1016/j.scitotenv.2023.162604 36878298 10.1016/j.scitotenv.2023.162604

[CR68] Gonçalves A, De Figueiredo D, Pereira M (2006) The effects of different salinity concentrations on growth of three freshwater green algae. Fresenius Environ Bull 15:1382–1386

[CR69] Gonçalves AL, Simões M, Pires JCM (2014) The effect of light supply on microalgal growth, CO_2_ uptake and nutrient removal from wastewater. Energy Convers Manag 85:530–536. 10.1016/j.enconman.2014.05.085

[CR70] Gonidakis S, Longo VD (2013) Assessing chronological aging in bacteria. Methods Mol Biol 965:421–437. 10.1007/978-1-62703-239-1_28 23296675 10.1007/978-1-62703-239-1_28 PMC4077615

[CR71] Guerinot ML, Yi Y (1994) Iron: nutritious, noxious, and not readily available. Plant Physiol 104:815–820. 10.1104/pp.104.3.815 12232127 10.1104/pp.104.3.815 PMC160677

[CR72] Guiry MD, Guiry GM (2015) *Raphidocelis subcapitata* (Korshikov) In: Nygaard K, Kristiansen J, Skulberg OM (eds) 1987. AlgaeBase. World-wide electronic publication. National University of Ireland, Galway. https://www.algaebase.org; accessed 26 May 2023

[CR73] Guo J, Zhang Y, Mo J, Sun H, Li Q (2021) Sulfamethoxazole-altered transcriptomein green alga *Raphidocelis subcapitata* suggests inhibition of translation and DNA damage repair. Front Microbiol 12:541451. 10.3389/fmicb.2021.541451 34349730 10.3389/fmicb.2021.541451 PMC8326373

[CR74] Gutierrez-Wing MT, Benson BC, Rusch KA (2012) Impact of light quality and quantity on growth rate kinetics of *Selenastrum capricornutum*. Eng Life Sci 12:79–88. 10.1002/elsc.201000217

[CR75] Hom-Diaz A, Llorca M, Rodríguez-Mozaz S, Vicent T, Barceló D, Blánquez P (2015) Microalgae cultivation on wastewater digestate: β-estradiol and 17α-ethynylestradiol degradation and transformation products identification. J Environ Manage 155:106–113. 10.1016/j.jenvman.2015.03.003 25785785 10.1016/j.jenvman.2015.03.003

[CR76] Hom-Diaz A, Jaén-Gil A, Rodríguez-Mozaz S, Barceló D, Vicent T, Blánquez P (2022) Insights into removal of antibiotics by selected microalgae (*Chlamydomonas reinhardtii*, *Chlorella sorokiniana*, *Dunaliella tertiolecta* and *Pseudokirchneriella subcapitata*). Algal Res 61:102560. 10.1016/j.algal.2021.102560

[CR77] Hughes EO, Gorham PR, Zehnder A (1958) Toxicity of a unialgal culture of *Microcystis aeruginosa*. Can J Microbiol 4:225–236. 10.1139/m58-024 13536907 10.1139/m58-024

[CR78] ISO (2012) ISO 8692/2012. Water quality—freshwater algal growth inhibition test with unicellular green algae. International Standards Organization. https://www.iso.org/standard/54150.html. Accessed 23 Jun 2023

[CR79] Jakubczak M, Bury D, Purbayanto MAK, Wójcik A, Moszczyńska D, Prenger K, Naguib M, Jastrzębska AM (2022) Understanding the mechanism of Nb-MXene bioremediation with green microalgae. Sci Rep 12:14366. 10.1038/s41598-022-18154-3 35999240 10.1038/s41598-022-18154-3 PMC9399251

[CR80] Kaur N, Chugh V, Gupta AK (2014) Essential fatty acids as functional components of foods- a review. J Food Sci Technol 51:2289–2303. 10.1007/s13197-012-0677-0 25328170 10.1007/s13197-012-0677-0 PMC4190204

[CR81] Kreutzer A, Faetsch S, Heise S, Hollert H, Witt G (2022) Passive dosing: assessing the toxicity of individual PAHs and recreated mixtures to the microalgae *Raphidocelis subcapitata*. Aquat Toxicol 249:106220. 10.1016/j.aquatox.2022.106220 35777163 10.1016/j.aquatox.2022.106220

[CR82] Krienitz L, Bock C (2012) Present state of the systematics of planktonic coccoid green algae of inland waters. Hydrobiologia 698:295–326. 10.1007/s10750-012-1079-z

[CR83] Krienitz L, Ustinova I, Friedl T, Huss VAR (2001) Traditional generic concepts versus 18S rRNA gene phylogeny in the green algal family *Selenastraceae* (*Chlorophyceae, Chlorophyta*). J Phycol 37:852–865. 10.1046/j.1529-8817.2001.01004.x

[CR84] Krienitz L, Bock C, Nozaki H, Wolf M (2011) SSU rRNA gene phylogeny of morphospecies affiliated to the bioassay alga *Selenastrum capricornutum* recovered the polyphyletic origin of crescent-shaped *Chlorophyta*. J Phycol 47:880–893. 10.1111/j.1529-8817.2011.01010.x 27020023 10.1111/j.1529-8817.2011.01010.x

[CR85] Lachmann SC, Mettler-Altmann T, Wacker A, Spijkerman E (2019) Nitrate or ammonium: influences of nitrogen source on the physiology of a green alga. Ecol Evol 9:1070–1082. 10.1002/ece3.4790 30805141 10.1002/ece3.4790 PMC6374670

[CR86] Lavoie M, Bernier J, Fortin C, Campbell PGC (2009) Cell homogenization and subcellular fractionation in two phytoplanktonic algae: implications for the assessment of metal subcellular distributions. Limnol Oceanogr Methods 7:277–286. 10.4319/lom.2009.7.277

[CR87] Lee RE (2018) Chlorophyta. Phycology, 5th edn. Cambridge University Press, Cambridge, pp 133–230

[CR88] Lee E, Jalalizadeh M, Zhang Q (2015) Growth kinetic models for microalgae cultivation: a review. Algal Res 12:497–512. 10.1016/j.algal.2015.10.004

[CR89] Lei A-P, Hu Z-L, Wong Y-S, Tam NF-Y (2007) Removal of fluoranthene and pyrene by different microalgal species. Bioresour Technol 98:273–280. 10.1016/j.biortech.2006.01.012 16517155 10.1016/j.biortech.2006.01.012

[CR90] Leischmann AA, Greene JC, Miller WE (1979) Bibliography of literature pertaining to the genus *Selenastrum*. United States Environmental Protection Agency, Freshwater Systems Division, Corvallis Environmental Research Laboratory, Corvallis, Oregon; EPA-600/9–79–02

[CR91] Lekamge S, Miranda AF, Abraham A, Ball AS, Shukla R, Nugegoda D (2020) The toxicity of coated silver nanoparticles to the alga *Raphidocelis subcapitata*. SN Appl Sci 2:596. 10.1007/s42452-020-2430-z

[CR92] Leliaert F, Smith DR, Moreau H, Herron MD, Verbruggen H, Delwiche CF, De Clerck O (2012) Phylogeny and molecular evolution of the green algae. CRC Crit Rev Plant Sci 31:1–46. 10.1080/07352689.2011.615705

[CR93] Lemieux C, Otis C, Turmel M (2014) Chloroplast phylogenomic analysis resolves deep-level relationships within the green algal class *Trebouxiophyceae*. BMC Evol Biol 14:211. 10.1186/s12862-014-0211-2 25270575 10.1186/s12862-014-0211-2 PMC4189289

[CR94] Li Q, Lu D, Sun H, Guo J, Mo J (2021) Tylosin toxicity in the alga *Raphidocelis subcapitata* revealed by integrated analyses of transcriptome and metabolome: photosynthesis and DNA replication-coupled repair. Aquat Toxicol 239:105964. 10.1016/j.aquatox.2021.105964 34534865 10.1016/j.aquatox.2021.105964

[CR95] Li J, Wang T, Xue J (2023) Toxic effects of disinfection by-products on *Pseudokirchneriella subcapitata* and co-cultured algae community. Sci Total Environ 894:164760. 10.1016/j.scitotenv.2023.164760 37343859 10.1016/j.scitotenv.2023.164760

[CR96] Liang Y, Beardall J, Heraud P (2006) Changes in growth, chlorophyll fluorescence and fatty acid composition with culture age in batch cultures of *Phaeodactylum tricornutum* and *Chaetoceros muelleri* (*Bacillariophyceae*). Bot Mar 49:165–173 10.1515/BOT.2006.021 10.1016/j.jphotobiol.2005.11.002 16388965

[CR97] Lichtenthaler K, Welburn AR (1983) Determination of total carotenoids and chlorophylls a and b of leaf extracts in different solvents. Biochem Soc Trans 11:591–592. 10.1042/bst0110591

[CR98] Liu X, Sun J, Wei Y, Liu Y (2023) Relationship between cell volume and particulate organic matter for different size phytoplankton. Mar Pollut Bull 194:115298. 10.1016/j.marpolbul.2023.115298 37499568 10.1016/j.marpolbul.2023.115298

[CR99] Lum K, Kim J, Lei X (2013) Dual potential of microalgae as a sustainable biofuel feedstock and animal feed. J Anim Sci Biotechnol 4:53. 10.1186/2049-1891-4-53 24359607 10.1186/2049-1891-4-53 PMC3881014

[CR100] Luo L, Xiao Z, Zhou X, Yang L, Luo S, Zhao C, Luan T (2020) Quantum chemical calculation to elucidate the biodegradation pathway of methylphenanthrene by green microalgae. Water Res 173:115598. 10.1016/j.watres.2020.115598 32062219 10.1016/j.watres.2020.115598

[CR101] Lürling M, Van Donk E (1997) Morphological changes in *Scenedesmus* induced by infochemicals released in situ from zooplankton grazers. Limnol Oceanogr 42:783–788. 10.4319/lo.1997.42.4.0783

[CR102] Machado MD, Soares EV (2012a) Development of a short-term assay based on the evaluation of the plasma membrane integrity of the alga *Pseudokirchneriella subcapitata*. Appl Microbiol Biotechnol 95:1035–1042. 10.1007/s00253-012-4185-y 22660770 10.1007/s00253-012-4185-y

[CR103] Machado MD, Soares EV (2012b) Assessment of cellular reduced glutathione content in *Pseudokirchneriella subcapitata* using monochlorobimane. J Appl Phycol 24:1509-1516. 10.1007/s10811-012-9811-7

[CR104] Machado MD, Soares EV (2013) Optimization of a microplate-based assay to assess esterase activity in the alga *Pseudokirchneriella subcapitata*. Water Air Soil Pollut 224:1358. 10.1007/s11270-012-1358-3

[CR105] Machado MD, Soares EV (2014) Modification of cell volume and proliferative capacity of *Pseudokirchneriella subcapitata* cells exposed to metal stress. Aquat Toxicol 147:1–6. 10.1016/j.aquatox.2013.11.017 24342441 10.1016/j.aquatox.2013.11.017

[CR106] Machado MD, Soares EV (2015a) Use of a fluorescence-based approach to assess short-term responses of the alga *Pseudokirchneriella subcapitata* to metal stress. J Appl Phycol 27:805–813. 10.1007/s10811-014-0351-1

[CR107] Machado MD, Soares EV (2015b) Quantification and viability analyses of *Pseudokirchneriella subcapitata* algal cells using image-based cytometry. J Appl Phycol 27:703–710. 10.1007/s10811-014-0377-4

[CR108] Machado MD, Soares EV (2019a) Impact of erythromycin on a non-target organism: cellular effects on the freshwater microalga *Pseudokirchneriella subcapitata*. Aquat Toxicol 208:179–186. 10.1016/j.aquatox.2019.01.014 30682620 10.1016/j.aquatox.2019.01.014

[CR109] Machado MD, Soares EV (2019b) Sensitivity of freshwater and marine green algae to three compounds of emerging concern. J Appl Phycol 31:399–408. 10.1007/s10811-018-1511-5

[CR110] Machado MD, Soares EV (2020) Reproductive cycle progression arrest and modification of cell morphology (shape and biovolume) in the alga *Pseudokirchneriella subcapitata* exposed to metolachlor. Aquat Toxicol 222:105449. 10.1016/j.aquatox.2020.105449 32109756 10.1016/j.aquatox.2020.105449

[CR111] Machado MD, Soares EV (2021a) Toxicological effects induced by the biocide triclosan on *Pseudokirchneriella subcapitata*. Aquat Toxicol 230:105706. 10.1016/j.aquatox.2020.105706 33302172 10.1016/j.aquatox.2020.105706

[CR112] Machado MD, Soares EV (2021b) Exposure of the alga *Pseudokirchneriella subcapitata* to environmentally relevant concentrations of the herbicide metolachlor: impact on the redox homeostasis. Ecotoxicol Environ Saf 207:111264. 10.1016/j.ecoenv.2020.111264 32911184 10.1016/j.ecoenv.2020.111264

[CR113] Machado MD, Soares EV (2022) Life and death of *Pseudokirchneriella subcapitata*: physiological changes during chronological aging. Appl Microbiol Biotechnol 106:8245–8258. 10.1007/s00253-022-12267-5 36385567 10.1007/s00253-022-12267-5

[CR114] Machado MD, Soares EV (2023) Palmelloid-like phenotype in the alga *Raphidocelis subcapitata* exposed to pollutants: a generalized adaptive strategy to stress or a specific cellular response? Aquat Toxicol 264:106732. 10.1016/j.aquatox.2023.106732 37879199 10.1016/j.aquatox.2023.106732

[CR115] Machado MD, Lopes AR, Soares EV (2015) Responses of the alga *Pseudokirchneriella subcapitata* to long-term exposure to metal stress. J Hazard Mater 296:82–92. 10.1016/j.jhazmat.2015.04.022 25913674 10.1016/j.jhazmat.2015.04.022

[CR116] Maltsev Y, Maltseva K, Kulikovskiy M, Maltseva S (2021) Influence of light conditions on microalgae growth and content of lipids, carotenoids, and fatty acid composition. Biology (basel) 10:1060. 10.3390/biology10101060 34681157 10.3390/biology10101060 PMC8533579

[CR117] Mansano AS, Moreira RA, Dornfeld HC, Freitas EC, Vieira EM, Sarmento H, Rocha O, Seleghim MHR (2017) Effects of diuron and carbofuran and their mixtures on the microalgae *Raphidocelis subcapitata*. Ecotoxicol Environ Saf 142:312–321. 10.1016/j.ecoenv.2017.04.024 28433596 10.1016/j.ecoenv.2017.04.024

[CR118] Marin B (2012) Nested in the *Chlorellales* or independent class? Phylogeny and classification of the *Pedinophyceae* (*Viridiplantae*) revealed by molecular phylogenetic analyses of complete nuclear and plastid-encoded rRNA operons. Protist 163:778–805. 10.1016/j.protis.2011.11.004 22192529 10.1016/j.protis.2011.11.004

[CR119] Matsumura K, Yagi T, Yasuda K (2003) Role of timer and sizer in regulation of *Chlamydomonas* cell cycle. Biochem Biophys Res Commun 306:1042–1049. 10.1016/S0006-291X(03)01089-1 12821148 10.1016/s0006-291x(03)01089-1

[CR120] McKeel E, Kim H-I, Jeon S-J, Giraldo JP, Klaper R (2024) The effect of nanoparticle surface charge on freshwater algae growth, reproduction, and lipid production. Environ Sci Nano. 10.1039/D3EN00353A

[CR121] McLean RJ (1968) Ultrastructure of *Spongiochloris typica* during senescence. J Phycol 4:277–283. 10.1111/j.1529-8817.1968.tb04696.x 27068200 10.1111/j.1529-8817.1968.tb04696.x

[CR122] Miller WE, Greene JC, Shiroyama T (1978) The *Selenastrum capricornutum* Printz algal assay bottle test: experimental design, application, and data interpretation protocol. (US-EPA-600/9–78–018), United States Environmental Protection Agency, Office of Research and Development, Environmental Research Laboratory, Report Number EPA-600/9–78–018, Corvallis, Ore

[CR123] Minguez L, Bureau R, Halm-Lemeille M-P (2018) Joint effects of nine antidepressants on *Raphidocelis subcapitata* and *Skeletonema marinoi*: a matter of amine functional groups. Aquat Toxicol 196:117–123. 10.1016/j.aquatox.2018.01.015 29367071 10.1016/j.aquatox.2018.01.015

[CR124] Meyer MT, Whittaker C, Griffiths H (2017) The algal pyrenoid: key unanswered questions. J Exp Bot 68:3739–3749. 10.1093/jxb/erx178 10.1093/jxb/erx178 28911054

[CR125] Mizukami-Murata S, Suzuki Y, Sakurai K, Yamashita H (2021) Freshwater alga *Raphidocelis subcapitata* undergoes metabolomic changes in response to electrostatic adhesion by micrometer-sized nylon 6 particles. Environ Sci Pollut Res 28:66901–66913. 10.1007/s11356-021-15300-8 10.1007/s11356-021-15300-8 PMC8642260 34236613

[CR126] Mo J, Qi Q, Hao Y, Lei Y, Guo J (2022) Transcriptional response of a green alga (*Raphidocelis subcapitata*) exposed to triclosan: photosynthetic systems and DNA repair. J Environ Sci 111:400–411. 10.1016/j.jes.2021.04.023 10.1016/j.jes.2021.04.023 34949369

[CR127] Mo J, Ma Z, Yan S, Cheung NKM, Yang F, Yao X, Guo J (2023) Metabolomic profiles in a green alga (*Raphidocelis subcapitata*) following erythromycin treatment: ABC transporters and energy metabolism. J Environ Sci 124:591–601. 10.1016/j.jes.2021.12.001 10.1016/j.jes.2021.12.001 36182165

[CR128] Moreira RA, Rocha GS, da Silva LCM, Goulart BV, Montagner CC, da Graça Maria, Melão Gama, Espindola Evaldo Luiz Gaeta (2020) Exposure to environmental concentrations of fipronil and 2,4-D mixtures causes physiological, morphological and biochemical changes in *Raphidocelis subcapitata*. Ecotoxicol Environ Saf 206:111180. 10.1016/j.ecoenv.2020.111180 32861006 10.1016/j.ecoenv.2020.111180

[CR129] Moreira BRA, Viana CRA, Cruz VH, Lopes PRM, da Silva VR, Ramos RAV (2022) Meta-analytic review on third-generation biodiesel. BioEnergy Res 15:27–45. 10.1007/s12155-020-10232-6

[CR130] Moreno-Garcia L, Adjallé K, Barnabé S, Raghavan GSV (2017) Microalgae biomass production for a biorefinery system: recent advances and the way towards sustainability. Renew Sustain Energy Rev 76:493–506. 10.1016/j.rser.2017.03.024

[CR131] Nagai T, Ishihara S, Yokoyama A, Iwafune T (2011) Effects of four rice paddy herbicides on algal cell viability and the relationship with population recovery. Environ Toxicol Chem 30:1898–1905. 10.1002/etc.582 21590715 10.1002/etc.582

[CR132] Nagappan S, Das P, AbdulQuadir M, Thaher M, Khan S, Mahata C, Al-Jabri H, Vatland AK, Kumar G (2021) Potential of microalgae as a sustainable feed ingredient for aquaculture. J Biotechnol 341:1–20. 10.1016/j.jbiotec.2021.09.003 34534593 10.1016/j.jbiotec.2021.09.003

[CR133] Nascimento IA, Marques SSI, Cabanelas ITD, Pereira SA, Druzian JI, de Souza CO, Vich DV, de Carvalho GC, Nascimento MA (2013) Screening microalgae strains for biodiesel production: lipid productivity and estimation of fuel quality based on fatty acids profiles as selective criteria. BioEnergy Res 6:1–13. 10.1007/s12155-012-9222-2

[CR134] Nascimento VM, Nascimento KM, Fonseca GG (2020) Biotechnological potential of *Pseudokirchneriella subcapitata*, *Scenedesmus spinous*, and *Scenedesmus acuminatus*. Acta Aliment 49:154–162. 10.1556/066.2020.49.2.4

[CR135] Nichols HW, Bold HC (1965) *Trichosarcina polymorpha* gen. et sp. nov. J Phycol 1:34–38. 10.1111/j.1529-8817.1965.tb04552.x

[CR136] NIES (2023) *Raphidocelis subcapitata* (Korshikov). In: Nygaard K, Kristiansen J, Skulberg OM. Strain no. NIES-35. Microbial Culture Colection. National Institute of Environmental Studies. https://mcc.nies.go.jp/strainList.do?strainId=26. Accessed 7 Jul 2023

[CR137] NORCCA (2023a) Raphidocelis *subcapitata*. Strain NIVA-CHL 1. The Norwegian Culture Collection of Algae. . https://norcca.scrol.net/strain/niva-chl-1. Accessed 2 Jun 2023

[CR138] NORCCA (2023b) Pricing. The Norwegian Culture Collection of Algae. https://norcca.scrol.net/basic-page/pricing. Accessed 10 Jul 2023

[CR139] OECD (2011) Test no. 201: Freshwater alga and cyanobacteria, growth inhibition test. Organization for Economic Cooperation and Development, Paris, France

[CR140] OECD-FAO (2023) OECD-FAO Agricultural Outlook (Edition 2022). Organisation for Economic Co-operation and Development (OECD) and Food and Agriculture Organization of the United Nations (FAO). https://www.oecd-ilibrary.org/content/data/13d66b76-en. Accessed 15 May 2023

[CR141] Ostovich E, Henke A, Green C, Laudadio E, Spehlmann M, Van Ee N, Uertz J, Hamers R, Klaper R (2023) Physiological impacts on *Raphidocelis subcapitata* in response to lithiated cobalt oxide nanomaterials. Environ Toxicol Chem 42:1451–1462. 10.1002/etc.5617 37036253 10.1002/etc.5617

[CR142] Ostovich E, Henke A, Green CM, Hamers RJ, Klaper R (2024) Predicting the phytotoxic mechanism of action of LiCoO2 nanomaterials using a novel multiplexed algal cytological imaging (MACI) assay and machine learning. Environ Sci Nano. 10.1039/D3EN00629H 10.1021/acs.est.3c07733 38446593

[CR143] Padmakumar V, Tharavathy N (2020) First identification of the chlorophyte algae *Pseudokirchneriella subcapitata* (Korshikov) Hindák in lake waters of India. Nat Environ Pollut Technol 19:409–412

[CR144] Pascual G, Sano D, Sakamaki T, Nishimura O (2020) Effects of chemical interaction of nutrients and EDTA on metals toxicity to *Pseudokirckneriella subcapitata*. Ecotoxicol Environ Saf 203:110966. 10.1016/j.ecoenv.2020.110966 32678755 10.1016/j.ecoenv.2020.110966

[CR145] Pascual G, Sano D, Sakamaki T, Akiba M, Nishimura O (2022) The water temperature changes the effect of pH on copper toxicity to the green microalgae *Raphidocelis subcapitata*. Chemosphere 291:133110. 10.1016/j.chemosphere.2021.133110 34848234 10.1016/j.chemosphere.2021.133110

[CR146] Patel M, Kumar R, Kishor K, Mlsna T, Pittman CUJ, Mohan D (2019) Pharmaceuticals of emerging concern in aquatic systems: chemistry, occurrence, effects, and removal methods. Chem Rev 119:3510–3673. 10.1021/acs.chemrev.8b00299 30830758 10.1021/acs.chemrev.8b00299

[CR147] Patil V, Wenner D, Mortensen L, Gislerod HR (2006) Effect of light quality on growth of *Nannochlorpsis oceancia* and *Pseudokirchneriella subcapitata* in bioreactors. Acta Horticulturae. International Society for Horticultural Science (ISHS), Leuven, Belgium, pp 207–212

[CR148] Patil V, Källqvist T, Olsen E, Vogt G, Gislerød HR (2007) Fatty acid composition of 12 microalgae for possible use in aquaculture feed. Aquac Int 15:1–9. 10.1007/s10499-006-9060-3

[CR149] Peng S, Long M, Zheng L, Song L, Li J (2019) Physiological sensitivity of *Haematococcus pluvialis* (*Chlorophyta*) to environmental pollutants: a comparison with *Microcystis wesenbergii* (*Cyanobacteria*) and *Pseudokirchneriella subcapitata* (*Chlorophyta*). J Appl Phycol 31:365–374. 10.1007/s10811-018-1557-4

[CR150] Peng J, Guo J, Lei Y, Mo J, Sun H, Song J (2021) Integrative analyses of transcriptomics and metabolomics in *Raphidocelis subcapitata* treated with clarithromycin. Chemosphere 266:128933. 10.1016/j.chemosphere.2020.128933 33223212 10.1016/j.chemosphere.2020.128933

[CR151] Pérez-Gálvez A, Viera I, Roca M (2020) Carotenoids and chlorophylls as antioxidants. Antioxidants (basel, Switzerland) 9:505. 10.3390/antiox9060505 32526968 10.3390/antiox9060505 PMC7346216

[CR152] Pinzi S, Dorado M (2012) Feedstocks for advanced biodiesel production. In: Luque R, Melero JA (eds) Advances in biodiesel production: processes and technologies. Woodhead Publishing Limited, Cambridge, UK, pp 69–90

[CR153] Pitre D, Boullemant A, Fortin C (2014) Uptake and sorption of aluminium and fluoride by four green algal species. Chem Cent J 8:8. 10.1186/1752-153X-8-8 24485034 10.1186/1752-153X-8-8 PMC3937126

[CR154] Pollio A, Pinto G, Ligrone R, Aliotta G (1993) Effects of the potential allelochemical α-asarone on growth, physiology and ultrastructure of two unicellular green algae. J Appl Phycol 5:395–403. 10.1007/BF02182732

[CR155] Priyanka P, Kinsella GK, Henehan GT, Ryan BJ (2020) Enzymatic in-situ transesterification of neutral lipids from simulated wastewater cultured *Chlorella emersonii* and *Pseudokirchneriella subcapitata* to sustainably produce fatty acid methyl esters. Bioresour Technol Reports 11:100489. 10.1016/j.biteb.2020.100489

[CR156] Quigg A (2008) Trace elements. In: Jørgensen SE, Fath BDBT-E of E (eds) Academic Press, Oxford, pp 3564–3573

[CR157] Reynolds JH, Middlebrooks EJ, Porcella DB, Grenney WJ (1975) Effects of temperature on growth constants of *Selenastrum capricornutum*. J Water Pollut Control Fed 47:2420–2436 1219144

[CR158] Reynolds A, Giltrap DM, Chambers PG (2021) Acute growth inhibition & toxicity analysis of nano-polystyrene spheres on *Raphidocelis subcapitata*. Ecotoxicol Environ Saf 207:111153. 10.1016/j.ecoenv.2020.111153 32896819 10.1016/j.ecoenv.2020.111153

[CR159] Ribeiro VR, Osório HDD, Ulrich AC, Rizzetti TM, Barrios AS, Barrios Andréa Sanchez, de Cassia Rosana, de Souza Schneider, Lisianne Brittes Benitez, (2022) The use of microalgae-microbial fuel cells in wastewater bioremediation and bioelectricity generation. J Water Process Eng 48:102882. 10.1016/j.jwpe.2022.102882

[CR160] Rioboo C, O’Connor JE, Prado R, Herrero C, Cid A (2009) Cell proliferation alterations in *Chlorella* cells under stress conditions. Aquat Toxicol 94:229–237. 10.1016/j.aquatox.2009.07.009 19679360 10.1016/j.aquatox.2009.07.009

[CR161] Rippka R, Deruelles J, Waterbury JB, Herdman M, Stanier RY (1979) Generic assignments, strain histories, and properties of pure cultures of cyanobacteria. Microbiology 111:1–61. 10.1099/00221287-111-1-1

[CR162] Rocha GS, Parrish CC, Espíndola ELG (2021) Effects of copper on photosynthetic and physiological parameters of a freshwater microalga (*Chlorophyceae*). Algal Res 54:102223. 10.1016/j.algal.2021.102223

[CR163] Rojíčková-Padrtová R, Maršálek B, Holoubek I (1998) Evaluation of alternative and standard toxicity assays for screening of environmental samples: selection of an optimal test battery. Chemosphere 37:495–507. 10.1016/S0045-6535(98)00065-4

[CR164] Russo C, Nugnes R, Orlo E, di Matteo A, De Felice B, Montanino C, Lavorgna M, Isidori M (2023) Diclofenac eco-geno-toxicity in freshwater algae, rotifers and crustaceans. Environ Pollut 335:122251. 10.1016/j.envpol.2023.122251 37506803 10.1016/j.envpol.2023.122251

[CR165] SAG (2023a) Culture maintenance. Culture Collection of Algae at the University of Göttingen, Germany. Sammlung von Algenkulturen (SAG). https://www.uni-goettingen.de/en/www.uni-goettingen.de/de/186227.html. Accessed 7 Jul 2023

[CR166] SAG (2023b) *Raphidocelis *subcapitata. Strain 61.81. Culture Collection of Algae at Gottingen University. Sammlung von Algenkulturen. https://sagdb.uni-goettingen.de/detailedList.php?str_number=61.81. Accessed 10 Jul 2023

[CR167] Saliu F, Magoni C, Torelli A, Cozza R, Lasagni M, Labra M (2021) Omega-3 rich oils from microalgae: a chitosan mediated in situ transesterification method. Food Chem 337:127745. 10.1016/j.foodchem.2020.127745 32795855 10.1016/j.foodchem.2020.127745

[CR168] Sato N, Toyoshima M (2021) Dynamism of metabolic carbon flow of starch and lipids in *Chlamydomonas debaryana*. Front Plant Sci 12:646498. 10.3389/fpls.2021.646498 33868347 10.3389/fpls.2021.646498 PMC8047662

[CR169] Sbrilli G, Calamati E, Boccalini S, Bimbi B, Pistolesi F (2003) Effects of nutrients and salinity on the algal assay using *Pseudokirchneriella subcapitata* (Korshikov) Hindak. Bull Environ Contam Toxicol 71:609–616. 10.1007/s00128-003-8633-3 14567589 10.1007/s00128-003-8633-3

[CR170] Shaikh S (2022) Sources and health benefits of functional food components. In: Savitskaya A (ed) Shiomi N. Current topics in functional food IntechOpen, Rijeka, pp 1–27

[CR171] Shetty P, Gitau MM, Maróti G (2019) Salinity stress responses and adaptation mechanisms in eukaryotic green microalgae. Cells 8:1657. 10.3390/cells8121657 31861232 10.3390/cells8121657 PMC6952985

[CR172] Silva NFP, Gonçalves AL, Moreira FC, Silva TFCV, Martins FG, Alvim-Ferraz MCM, Boaventura RAR, Vilar VJP, Pires JCM (2015) Towards sustainable microalgal biomass production by phycoremediation of a synthetic wastewater: a kinetic study. Algal Res 11:350–358. 10.1016/j.algal.2015.07.014

[CR173] Skulberg OM (1964) Algal problems related to the eutrophication of European water supplies, and a bio-assay method to assess fertilizing influences of pollution on inland waters. In: Jackson DF (ed) Algae and man. Springer, Boston, MA, pp 262–299

[CR174] Smith AD (2000) Oxford dictionary of biochemistry and molecular biology, 2nd edn. Oxford University Press, Oxford, UK, p 523

[CR175] Sousa CA, Soares HMVM, Soares EV (2018) Toxic effects of nickel oxide (NiO) nanoparticles on the freshwater alga *Pseudokirchneriella subcapitata*. Aquat Toxicol 204:80–90. 10.1016/j.aquatox.2018.08.022 30205248 10.1016/j.aquatox.2018.08.022

[CR176] Sousa CA, Soares HMVM, Soares EV (2019) Chronic exposure of the freshwater alga *Pseudokirchneriella subcapitata* to five oxide nanoparticles: hazard assessment and cytotoxicity mechanisms. Aquat Toxicol 214:105265. 10.1016/j.aquatox.2019.105265 31416018 10.1016/j.aquatox.2019.105265

[CR177] Stansell GR, Gray VM, Sym SD (2012) Microalgal fatty acid composition: implications for biodiesel quality. J Appl Phycol 24:791–801. 10.1007/s10811-011-9696-x

[CR178] Stirk WA, Ördög V, Novák O, Rolčík J, Strnad M, Bálint P, van Staden J (2013) Auxin and cytokinin relationships in 24 microalgal strains. J Phycol 49:459–467. 10.1111/jpy.12061 27007035 10.1111/jpy.12061

[CR179] Suzuki S, Yamaguchi H, Nakajima N, Kawachi M (2018) *Raphidocelis subcapitata* (=*Pseudokirchneriella subcapitata*) provides an insight into genome evolution and environmental adaptations in the Sphaeropleales. Sci Rep 8:8058. 10.1038/s41598-018-26331-6 29795299 10.1038/s41598-018-26331-6 PMC5966456

[CR180] Tippery NP, Fučíková K, Lewis PO, Lewis LA (2012) Probing the monophyly of the *Sphaeropleales* (*Chlorophyceae*) using data from five genes. J Phycol 48:1482–1493. 10.1111/jpy.12003 27009998 10.1111/jpy.12003

[CR181] US-EPA (1971) Algal assay procedure: bottle test. United States Environmental Protection Agency. National Eutrophication Research Program, Corvallis, Oregon, USA. Environmental Protection Agency, National Eutrophication Research Program

[CR182] US-EPA (1991) Technical support document for water-quality-based toxics control. United States Environmental Agency, United States

[CR183] US-EPA (2002) Methods for measuring the acute toxicity of effluents and receiving waters to freshwater and marine organisms. EPA-821-R-02–012, 5th edn. United States Environmental Protection Agency, Washington, DC

[CR184] US-EPA (2012) Algal toxicity (OCSPP 850.4500). Ecological effects test guidelines. Office of Chemical Safety and Pollution Prevention. United States Environmental Protection Agency. EPA 712-C-006, Washington, DC

[CR185] US-EPA (2023a) ECOTOX Knowledgebase. https://cfpub.epa.gov/ecotox/search.cfm. Accessed 10 Jul 2023

[CR186] US-EPA (2023b) Economics of biofuels. United States Environmental Protection Agency. https://www.epa.gov/environmental-economics/economics-biofuels. Accessed 28 Apr 2023

[CR187] UTEX (2023a) Selenastrum capricornutum. Strain UTEX 1648. Culture Collection of Algae at the University of Texas at Austin. https://utex.org/products/utex-1648?variant=30991927640154#details. Accessed 10 Jul 2023

[CR188] UTEX (2023b) Bold 3N medium composition. Culture Collection of Algae. The University of Texas at Austin. http://web.biosci.utexas.edu/utex/Media%20PDF/modified-bolds-3n-medium.pdf. Accessed 19 Jul 2023

[CR189] UTEX (2023c) Cryopreservation of microalgae. The University of Texas at Austin. https://utex.org/pages/cryopreservation. Accessed 7 Jul 2023

[CR190] Venâncio C, Anselmo E, Soares A, Lopes I (2017) Does increased salinity influence the competitive outcome of two producer species? Environ Sci Pollut Res 24:5888–5897. 10.1007/s11356-016-8346-x 10.1007/s11356-016-8346-x 28064393

[CR191] Venâncio C, Monteiro B, Lopes I, Sousa ACA (2023) Assessing the risks of capecitabine and its active metabolite 5-fluorouracil to freshwater biota. Environ Sci Pollut Res 30:58841–58854. 10.1007/s11356-023-26505-4 10.1007/s11356-023-26505-4 PMC10163094 36997780

[CR192] Vítová M, Zachleder V (2005) Points of commitment to reproductive events as a tool for analysis of the cell cycle in synchronous cultures of algae. Folia Microbiol 50:141–149. 10.1007/BF02931463 16110919 10.1007/BF02931463

[CR193] Wang XD, Lu YC, Xiong XH, Yuan Y, Lu LX, Liu YJ, Mao JH, Xiao WW (2020) Toxicological responses, bioaccumulation, and metabolic fate of triclosan in *Chlamydomonas reinhardtii*. Environ Sci Pollut Res Int 27:11246–11259. 10.1007/s11356-020-07704-9 31960244 10.1007/s11356-020-07704-9

[CR194] Wang S, Mukhambet Y, Esakkimuthu S, Abomohra AE-F (2022) Integrated microalgal biorefinery – routes, energy, economic and environmental perspectives. J Clean Prod 348:131245. 10.1016/j.jclepro.2022.131245

[CR195] Wang P, Luan J, Luo L (2023) Removal of estrogens from primary settled sewage by repeated culture of *Selenastrum capricornutum*. Water Sci Technol 88:2837–2848. 10.2166/wst.2023.390 38096072 10.2166/wst.2023.390

[CR196] Watanabe MM (2005) Freshwater culture media. In: Andersen RA (ed) Algal culturing techniques. Elsevier Academic Press, Boston, pp 13–20

[CR197] Weiner JA, DeLorenzo ME, Fulton MH (2004) Relationship between uptake capacity and differential toxicity of the herbicide atrazine in selected microalgal species. Aquat Toxicol 68:121–128. 10.1016/j.aquatox.2004.03.004 15145222 10.1016/j.aquatox.2004.03.004

[CR198] Wu M, Wu G, Lu F, Wang H, Lei A, Wang J (2022) Microalgal photoautotrophic growth induces pH decrease in the aquatic environment by acidic metabolites secretion. Biotechnol Biofuels Bioprod 15:115. 10.1186/s13068-022-02212-z 36289523 10.1186/s13068-022-02212-z PMC9608927

[CR199] Yamagishi T, Yamaguchi H, Suzuki S, Horie Y, Tatarazako N (2017) Cell reproductive patterns in the green alga *Pseudokirchneriella subcapitata* (=*Selenastrum capricornutum*) and their variations under exposure to the typical toxicants potassium dichromate and 3,5-DCP. PLoS ONE 12:e0171259. 10.1371/journal.pone.0171259 28152022 10.1371/journal.pone.0171259 PMC5289587

[CR200] Yamagishi T, Yamaguchi H, Suzuki S, Yoshikawa M, Jameson I, Lorenz M, Nobles DR, Campbell C, Seki M, Kawachi M, Yamamoto H (2020) Comparative genome analysis of test algal strain NIVA-CHL1 (*Raphidocelis subcapitata*) maintained in microalgal culture collections worldwide. PLoS ONE 15:e0241889. 10.1371/journal.pone.0241889 33166324 10.1371/journal.pone.0241889 PMC7652255

[CR201] Yang L-H, Ying G-G, Su H-C, Stauber JL, Adams MS, Binet MT (2008) Growth-inhibiting effects of 12 antibacterial agents and their mixtures on the freshwater microalga *Pseudokirchneriella subcapitata*. Environ Toxicol Chem 27:1201–1208. 10.1897/07-471.1 18419195 10.1897/07-471.1

[CR202] Yee W (2016) Microalgae from the *Selenastraceae* as emerging candidates for biodiesel production: a mini review. World J Microbiol Biotechnol 32:64. 10.1007/s11274-016-2023-6 26931604 10.1007/s11274-016-2023-6

[CR203] Zamzam G, Lee CWJ, Milne F, Etsell J, Durnford DG (2022) Live long and prosper: acetate and its effects on longevity in batch culturing of *Chlamydomonas reinhardtii*. Algal Res 64:102676. 10.1016/j.algal.2022.102676

[CR204] Živković S, Veljković M (2018) Environmental impacts the of production and use of biodiesel. Environ Sci Pollut Res 25:191–199. 10.1007/s11356-017-0649-z 10.1007/s11356-017-0649-z 29124645

