# Supplementary material for: Features of the microalga Raphidocelis subcapitata: physiology and applications
Source: Appl Microbiol Biotechnol. 2024 Feb 19;108(1):219. doi: 10.1007/s00253-024-13038-0 (PMC10876740; doi:10.1007/s00253-024-13038-0)
Supplement: Supplementary file 1 — Supplementary file1 (PDF 116 KB) [file 253_2024_13038_MOESM1_ESM.pdf]

Applied Microbiology and Biotechnology

Supplementary Material

Features of the microalga *Raphidocelis subcapitata:* physiology and applications

Manuela D. Machado and Eduardo V. Soares[*]

Bioengineering Laboratory - CIETI, ISEP, Polytechnic of Porto, rua Dr António Bernardino de Almeida, 431, 4249-015 Porto, Portugal

CEB - Centre of Biological Engineering, University of Minho, Campus de Gualtar, 4710-057 Braga, Portugal

LABBELS - Associate Laboratory, Braga/Guimarães, Portugal

Manuela D. Machado ORCID: 000-0003-3519-8654

Eduardo V. Soares ORCID: 000-0003-2280-5291

CONTENT

Figure S1. Evolution of the works published with the topic "*Raphidocelis subcapitata*" or "*Chlamydomonas reinhardtii*" S2

[*]Corresponding author email address: evs@isep.ipp.pt

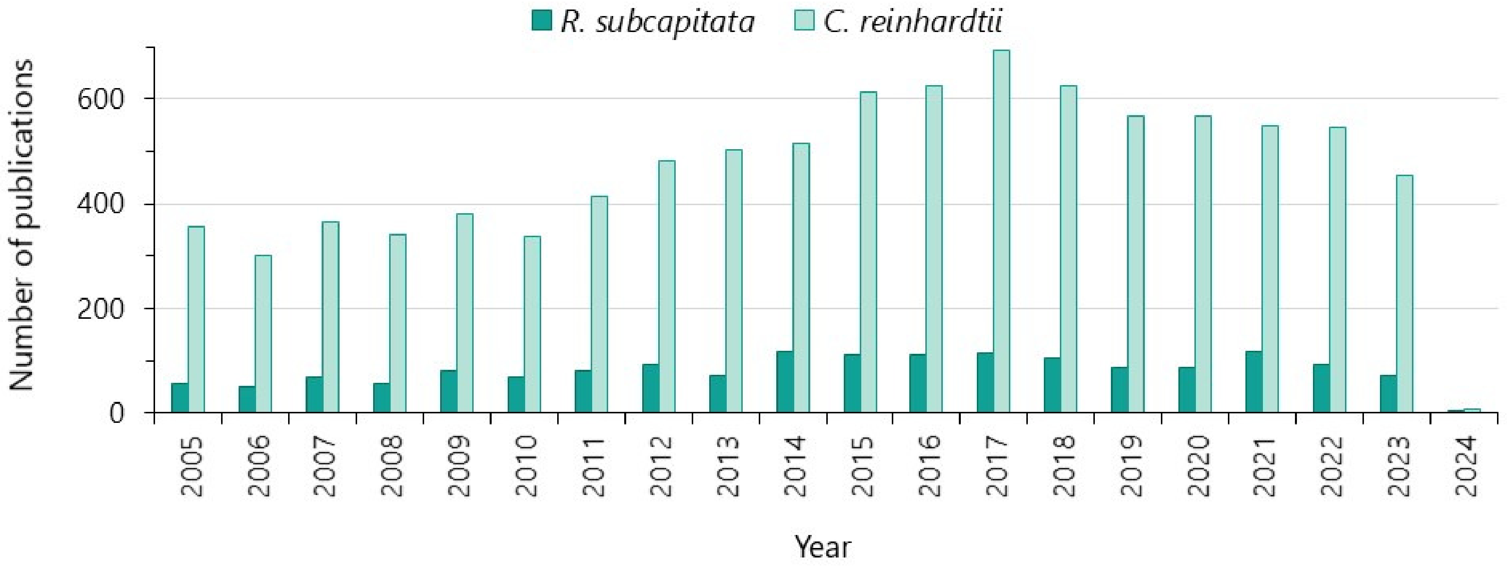

Fig. S1 Evolution of the works published with the topic "*Raphidocelis subcapitata* or *Pseudokirchneriella subcapitata* or *Selenastrum capricornutum*" (grouped in the graphic as *R. subcapitata*) or "*Chlamydomonas reinhardtii*". Source: Web of Science-Clarivate Analytics, "all fields", from 2005 to 2024; search: 10 January 2024
